# Demethylase ALKBH5 suppresses invasion of gastric cancer via PKMYT1 m6A modification

**DOI:** 10.1186/s12943-022-01522-y

**Published:** 2022-02-03

**Authors:** Yiyang Hu, Chunli Gong, Zhibin Li, Jiao Liu, Yang Chen, Yu Huang, Qiang Luo, Sumin Wang, Yu Hou, Shiming Yang, Yufeng Xiao

**Affiliations:** 1 Department of Gastroenterology, Xinqiao Hospital, Third Military Medical University, Chongqing, 400037 China; 2 Department of Endoscope, General Hospital of Northern Theater Command, Shenyang, Liaoning, 110016 China; 3 Department of Hematology, Southwest Hospital, Third Military Medical University, Chongqing, 400038 China; 4 Institute of Life Sciences, Chongqing Medical University, Chongqing, 400016 China

**Keywords:** ALKBH5, Invasion, Metastasis, Demethylase activity, PKMYT1, Gastric cancer

## Abstract

**Background:**

Gastric cancer (GC) is one of the most pernicious tumors that seriously harm human healthcare. GC metastasis is one of the prime cause of failed cancer treatment, but correlation between N6-methyladenosine (m6A) and GC metastasis was less reported.

**Methods:**

Methylated RNA immunoprecipitation sequencing (MeRIP-seq) of GC tissues was conducted. Quantitative real-time PCR (qRT-PCR), western blotting and immunohistochemistry (IHC) were taken to determine the expression of ALKBH5 in GC tissues and cell lines. RNA-seq together with MeRIP-qRT-PCR was used to screen the target gene of ALKBH5. RNA pulldown, mass spectrometry and RNA immunoprecipitation (RIP) were used to search the “reader” protein of target gene. The mechanism was also validated via a tail vein injection method for lung metastasis model.

**Results:**

Decreased expression of ALKBH5 was detected in GC samples, and it was correlated with clinical tumor distal metastasis and lymph node metastasis. ALKBH5 interference promoted metastasis of GC cells and this effect was closely related to the demethylase activity of ALKBH5. PKMYT1, as a downstream target of ALKBH5, promoted invasion and migration in GC. Caused by ALKBH5 knockdown or its demethylase activity mutation, upregulated expression of PKMYT1 indicated that ALKBH5 modulates expression of PKMYT1 in an m6A-dependent manner. IGF2BP3 helped stabilize the mRNA stability of PKMYT1 via its m6A modification site.

**Conclusions:**

This study established an ALKBH5-PKMYT1-IGF2BP3 regulation system in metastasis, representing a new therapeutic target for GC metastasis.

**Supplementary Information:**

The online version contains supplementary material available at 10.1186/s12943-022-01522-y.

## Background

Gastric cancer (GC) is the fifth most universal cancer type and ranks third among causes of cancer-related death in the world [1, 2]. Metastatic ability of GC cells is important cause of death. At present, treatment of patients with GC primarily depends on surgery, chemotherapy, biological therapy, and so on, but these methods remain unsatisfactory for patients and doctors [3]. The invasion and metastasis of GC is a persistent problem complicating current clinical treatment. Recent literature has revealed that the epigenetic changes in GC are closely related to their invasion and metastasis abilities [4, 5]. Therefore, an in-depth understanding of the epigenetic modification during metastatic process of GC cells has greatly aided in clinical treatment.

RNA modification, a form of epigenetic regulation, has been found to exist widely at the transcriptome level. As the most frequently modified form of eukaryotic mRNA, N6-methyladenosine (m6A) has been found to participate in a variety of biological processes [6, 7]. M6A is a reversible dynamic RNA modification regulated by m6A WERs (“writers”, “erasers” and “readers”) [8]. The fate of mRNA is altered differently by changes in m6A modifications in different regions of the mRNA. M6A modification of the 5'UTR of mRNA participates in mRNA splicing, stability, degradation, and polyadenylation, while modification of the 3 'UTR contributes to the nuclear export, translation efficiency, and maintenance of the structural stability of mRNA [9–14].

The role of m6A in diverse types of cancer has been reported recently [15, 16]. Publications have suggested that altered m6A modifications are widely participated in the process of tumor progression in various tumors, including GC [15, 17]. As an important epitranscriptome modulator, methyltransferase-like 3 (METTL3) has been reported to promote tumor angiogenesis and glycolysis by regulating HDGF m6A modification in GC [18]. Zinc finger MYM-type containing 1 (ZMYM1) was also recognized as an m6A target of METTL3 to facilitate metastasis by recruiting a complex to mediate the expression of E-cadherin [17]. SPHK2 and MYC are also regulated by METTL3 to potentiate migration and invasion [19, 20]. As a dynamic regulated system, the whole level of m6A modification was co-regulated by its “writers” and “erasers”. Most of these studies examined the mechanism of GC cell invasion and metastasis from the perspective of methyltransferases, but little is known regarding the involvement of demethylases. The specific molecular mechanism of demethylase involved in the invasion and metastasis of GC cells has not been fully clarified.

In this research, we identified the inhibitory effect of alkB homolog 5 (ALKBH5), one of the key demethylases, on the metastatic ability of GC cells. We further screened the key downstream molecules and modification sites and revealed the potential mechanism by which ALKBH5 regulates the invasion of GC cells.

## Methods

### GC patient samples

GC tissues and adjacent normal tissues from 49 GC patients were acquired from the Department of Surgery of Xinqiao Hospital and patients were diagnosed as GC by the departments of Pathology in hospital. Samples were quickly placed in liquid nitrogen for refrigeration. Informed consent for this procedure was gained from each participant.

### Cell culture

The human GC cell lines used were purchased from ATCC. HGC-27, BGC-823 and SGC-7901 GC cells were grown in DMEM medium with 10% FBS (Gibco) and 1% Penicillin–Streptomycin (Beyotime, China). Cells were identified with STR profiles. Mycoplasma decontamination was conducted once every two months.

### Immunoblotting

Cell pellets were charged at specific times and washed twice with PBS. Cell extracts were cleavage in RIPA (Beyotime, China). After 30 min of centrifugation at 12,000 × g, the supernatant was quantified using bicinchoninic acid (BCA) method (Beyotime, China). After quantification, sample was boiled at 100℃ with SDS buffer for 5 min. Protein extractions were separated using 10% or 12% SDS/PAGE gels. After incubated with primary and indicated HRP-conjugated secondary antibodies (Thermo Fisher Scientific, USA), membrane signals were exposed by chemiluminescence system (Bio-Rad, USA). Antibodies used were as follows: Anti-ALKBH5 (1:1000, Abcam, USA), Anti-GAPDH (1:10,000, GeneTex, USA), Anti-PKMYT1 (1:1000, Cell Signaling Technology, USA), Anti-IGF2BP3 (1:1000, Proteintech, USA).

### Constructs and transfections

Stable interference and overexpression of ALKBH5 lentiviruses were generated by GenePharma (Shanghai, China). PcDNA3.1 vector were used for the construction of the full-length PKMYT1-CDS, CDS-mut1, CDS-mut2, ALKBH5, and H204A plasmids, The ALKBH5-H204A plasmid and its lentiviruses were obtained from Genechem (Shanghai, China). Small interfering RNAs (siRNAs) targeting PKMYT1 and IGF2BP3 were directly synthesized (Ribobio, China). Transfection of plasmids or siRNAs was conducted using Lipofectamine 8000 (Beyotime, China). Cells were collected for further experiments two days after transfection. Sequences are listed in the Additional file 7.

### Animal models

After randomly assignment and anesthetization, nude mice were injected with 5 × 10^6^ cells suspended in 100 μl PBS into the tail vein (*n* = 5 per group). A Bruker Molecular Imaging Software (MA, USA) was used for monitoring metastatic progression 10 min after intraperitoneal injection (150 mg/kg) of D-Luciferin, Potassium Salt D (Genomeditech, China) dissolved in DMSO. The luciferase signal intensity was kept on the same scale. Mice were executed after 6 weeks and lungs were removed for immunohistochemical analysis and hematoxylin–eosin (H&E) staining. All operations were in line with laboratory animal management norms.

### RNA extraction, quantitative real-time PCR (qRT-PCR) and RNA-seq

TRIzol reagent (Invitrogen, USA) was used for RNA extraction. RNA was quantified using a Nanodrop 2000. PrimeScriptTM RT reagent kit (TAKARA, China) and TB GREEN SuperMix (TAKARA, China) were used for reverse transcribed and the qRT-PCR reactions to analyze mRNA expression. GAPDH was regarded as internal standard control to normalize the data. Experiment was repeated three times. For RNA-seq, RNA samples were sequenced by Lianchuan (Hangzhou, China) and analyzed using the OmicStudio tools at https://www.omicstudio.cn/tool.

### Methylated RNA immunoprecipitation sequencing (MeRIP-seq) and MeRIP-qRT-PCR

MeRIP was performed as formerly described [21] with minor modifications [22, 23]. Briefly, RNA was extracted and purified to deplete the ribosomal RNA and avoid DNA contamination. After fragmentation and denaturation, RNA was sheared into approximately 100-nt fragments and then incubated with an anti-m6A antibody (Abcam, USA) together with protein A/G magnetic beads (Thermo Scientific, USA) in immunoprecipitation buffer (150 mM NaCl, 10 mM Tris–HCl, pH 7.4, 0.1% NP-40) at 4℃ overnight. RNase inhibitor was also added. Antibody-combined methylated RNA was eluted with m6A and purified for further MeRIP sequencing by Novogene (Beijing, China). To examine m6A modification on individual genes, MeRIP-qRT-PCR was performed using the same procedures, except that RNA was sheared into approximately 200-nt fragments. One-tenth of the fragmented RNA was saved as a standardized control of input. Further m6A enrichment was calculated using qRT-PCR by normalizing to the input. HECBPA was used as a positive control for m6A modification.

### MeRIP-seq data analysis

After the samples qualified, FastQC software was used to perform basic quality statistics on the raw sequencing data (raw reads) and obtain high quality reads. BWA (Burrows Wheeler Aligner) was used for more accurate alignment of reads to the reference genome [24]. The reads with MAPQ (Mapping Quality) greater than 13 were regarded as the only reads to be compared for subsequent analysis. The distribution of reads on the functional regions of genes (exons, introns, 2 kb upstream of genes, 2 kb downstream of genes) was counted, and the exons and introns of each gene and 2 kb upstream and downstream of genes were divided into 100 bins each, and the number of reads falling into each bin was counted, and the number of reads in each bin was calculated as a percentage of the total number of reads in these regions. as the reads density of each bin. For a specific binding site, there is a significant enrichment of reads at its binding site. For single-end sequencing, MACS2 software was used to predict the fragment size (insert fragment) for IP experiments. MACS scans the genome with a certain window size, counts the enrichment of reads in each window, and then samples 1000 suitable windows to build an enrichment model to predict the length of inserted fragments. The predicted insert fragments were used for the subsequent peak analysis. Motif analysis was carried out to decide the specificity of IP and the confidence of the analysis, which efficiently predict related genes. Illumina Hiseq 2500 was used to perform sequencing with single-end 50-base pair(bp) read length.

### RNA immunoprecipitation (RIP)

The Magna RIP™ RNA-Binding Protein Immunoprecipitation Kit (Millipore, USA) was used. Briefly, protein A/G magnetic beads conjugated with rabbit immunoglobulin G (17–700, Millipore), ALKBH5 (Proteintech, USA) or IGF2BP3 (Proteintech, USA) antibody were incubated with cell lysates supplemented with RNase inhibitor at 4 °C overnight. After washed for 6 times, RNA–protein complexes were added into proteinase K buffer. Finally, RNA was extracted using phenol–chloroform method. QPCR was performed to determine relative interaction between ALKBH5 or IGF2BP3 protein and PKMYT1 transcripts.

### M6A RNA methylation assay

Total RNA of samples was extracted. EpiQuik m6A RNA Methylation Quantification Kit (Epigentek, USA) was taken to evaluate the global m6A levels of the mRNA. Briefly, 200 ng poly-A-purified RNAs were added to each assay well, and the relevant antibody were added to each well in a suitable diluted concentration, respectively. The OD450 was measured of each well. Quantification was performed according to the standard curve to calculate m6A levels.

### Gene ontology (GO) analysis and gene set enrichment analysis (GSEA)

GO analysis and GSEA were performed using DAVID (http://david.abcc.ncifcrf.gov/), the OmicStudio tools at https://www.omicstudio.cn/tool, and Cytoscape 3.7.0. *P* < 0.05 was considered statistically significant.

### Tissue microarray (TMA) and immunohistochemistry (IHC)

Two slides of Human Gastric Cancer TMA were purchased from Shanghai Outdo Biotech Company with additional ethical approval (China). TMAs were deparaffinized, subjected to antigen retrieval, and incubated with antibodies against ALKBH5 (1:500), PKMYT1 (1:500), and IGF2BP3 (1:400). Upright microscope system (Nikon, JAPAN) was used for images capturing. The stained slides were assessed with integrated optical density (IOD) using Image-Pro Plus software.

### Migration and invasion assays

For the transwell assay, chambers were present in a 24-well culture table with 800 ul DMEM containing 10% FBS prepared at the bottom. Then, 5 × 10^4^ cells in 200 ul serum-free medium were added into the upper layer. After 24 h, migrated cells in the lower layer were fixed, stained with crystal violet, and imaged for quantification. Pre-colded matrigel (Corning, USA) were added into the upper layer for the invasion assay.

### Wound healing assay

GC cells were plated into 24-well plates and incubated for 24–48 h until a 100% growth. Then, 200 ul micropipette tips were used to generate wounds. Next, cells were washed twice with 500 ul PBS. Then, 500 ul serum-free medium was added to the well. Photos were captured every 6 h for 24 h. Images were analyzed using ImageJ software.

### RNA stability assay

GC cells were grown into 12-well plates. Actinomycin D (5 μg/mL, Cell Signaling Technology, USA) was adopted to the plates. Cells were collected at the constant times for RNA extraction. The remaining PKMYT1 was analyzed by qRT-PCR. MRNA half-life was calculated using linear regression analysis. GAPDH was used for normalization.

### RNA pulldown and mass spectrometry analysis

Biotin-labeled PKMYT1 ssRNA probes were synthesized in vitro by Sangon Biotin (Shanghai, China), while the PKMYT1-CDS, CDS-mut1 and CDS-mut2 mRNA were first transcribed using the MEGAscript T7 Transcription Kit (Thermo Scientific, USA), and then biotin-labelled using Pierce RNA 3′ End Desthiobiotinylation Kit (Thermo Scientific, USA). Next, 20 pmol of biotinylated RNA together with cell lysate was mixed with streptavidin agarose beads (Thermo Scientific, USA) at 4℃ overnight. After six washes, streptavidin beads were collected for western blot or mass spectrometry.

### Statistical analysis

Statistics were analyzed using GraphPad Prism 9 (GraphPad Software, USA). Two-tailed unpaired student’s t-test or one-way ANOVA analysis was adopted. The Spearman/Pearson correlation analysis was used to evaluate relationships between ALKBH5 expression, PKMYT1 and IGF2BP3. Survival curves of GC patients were performed using Kaplan–Meier analysis. Receiver operating characteristic (ROC) curves were generated by GraphPad Prism 9. Each experiment has a minimum of three replications.

## Results

### MeRIP-seq revealed a strong correlation between m6A-modified genes and GC cell adhesion, and downregulation of demethylase ALKBH5 is correlated with GC prognosis

**Fig. 1:**
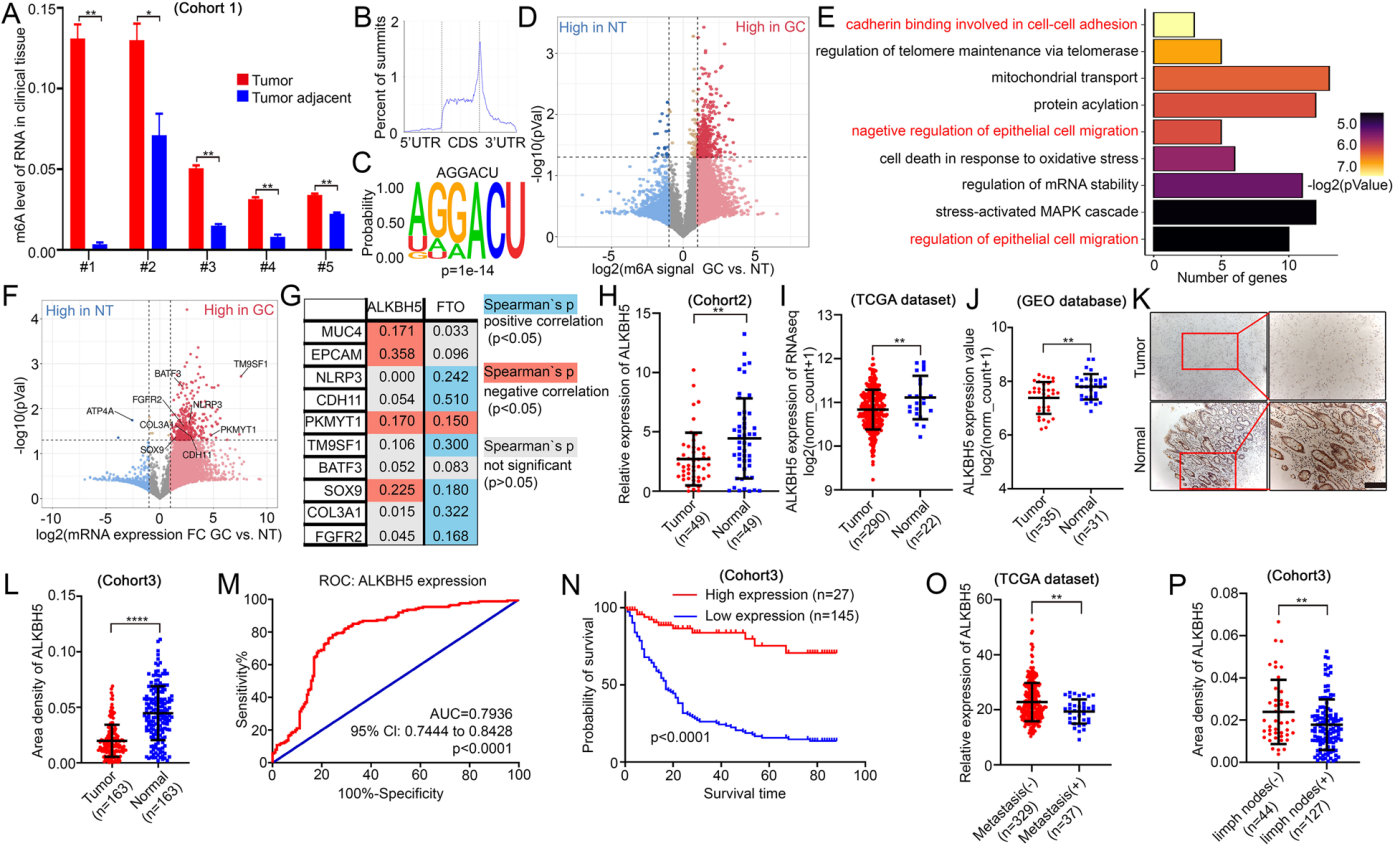
MeRIP-seq reveals correlation between m6A and cell adhesion, and ALKBH5 is associated with GC prognosis (**A**) M6A level of mRNA from 5 pairs of clinical tumor tissues with adjacent normal samples. (**B**) Peak distribution of m6A modification in meRIP-seq results. (**C**) The sequence motif identified from sequencing profile. (**D**) Volcano plot of m6A peaks detected by meRIP-seq in normal tissues (NT) and GC tissues. Red dots mean m6A peaks high in GC, while blue dots mean m6A peaks high in NT. Note that multiple peaks may map to the same gene. (**E**) GO analysis of genes with high m6A level in GC. (**F**) Volcano plot of mRNA level detected by meRIP-seq between NT and GC tissues. Red dots mean high expression of mRNA in GC, while blue dots mean high mRNA expression in NT. (**G**) Correlation analysis between metastatic-associated genes and m6A “eraser” in TCGA database. Blue means positive correlation (p < 0.05) and orange means negative correlation (p < 0.05). Grey means no significance (p > 0.05). (**H-J**) MRNA expression comparison of ALKBH5 between tumor and normal tissues among cohort2, TCGA database and GEO dataset. (**K-L**) Representative IHC pictures of ALKBH5 in GC tissue microarray (TMA) and the comparison of area density in ALKBH5-staining (scale bars = 100 µm). (**M**) The receiver operating characteristic (ROC) analysis of ALKBH5 expression in TMA. (**N**) Survival analysis of ALKBH5 expression in GC patients (p < 0.0001, log-rank test). (**O-P**) Expression comparison of ALKBH5 between metastasis-/ + and limph nodes metastasis-/ + group in TCGA dataset and TMA

To assess overall levels of m6A modification in GC, 5 pairs of clinical tumor tissues with adjacent normal samples were randomly selected. Using the EpiQuik m6A RNA methylation quantification kit, we observed significantly higher levels of m6A in GC tissues than in adjacent normal tissues (Fig. 1A), indicating that m6A might participate in the occurrence or progression of GC. Three pairs of tissues were selected for further Methylated RNA immunoprecipitation sequencing (MeRIP-seq). The m6A antibody-enriched RNA sequences were directly sequenced by high-throughput sequencing, and peak calling and the distribution of peaks were performed using MACS2 software [25] (with a threshold of q value = 0.05). Consistent with previous reports, m6A signal occurred primarily near the stop codon and the 3 'UTR of the mRNA transcript in the MeRIP-seq analysis of three pairs of tissues. (Fig. 1B). HOMER software was used to identify the motif (which indicates the sequence conservation of the m6A site) on the mRNA region bound by m6A peak. M6A modification was concentrated on the GGACU motif (Fig. 1C). Most genes exhibited higher levels of m6A modification and mRNA expression in tumor tissues (Fig. 1D, S1A-B). GO analysis indicated that these genes were primarily enriched in cell–cell adhesion and epithelial cell migration (Fig. 1E). KEGG analysis also showed tight junction and endosomal membrane enrichment (Fig. S1C). Global gene set enrichment analysis (GSEA) analysis revealed obviously enrichment in the phosphatidylinositol signaling system and RNA polymerase (Fig. S1D-F). Most genes that exhibited high m6A levels in tumor tissues also exhibited significantly higher mRNA levels than adjacent normal tissues as determined by data analysis (Fig. 1F).

M6A levels are primarily balanced by methyltransferases (METTL3, METTL14 and WTAP) and demethylases (FTO and ALKBH5), while methyltransferases METTL3 and METTL14 have been reported to regulate GC progression [18, 26]. Nevertheless, the mechanism of demethylation in GC is less reported. To explore the key demethylase that participates in cell migration, the correlation between m6A-related enzymes and migration-related genes was explored using the TCGA database. The data revealed a significant negative association of ALKBH5 with these genes, while FTO was primarily positively associated with them (Fig. 1G). Detection of mRNA levels in 49 patients' GC tissues and normal tissues suggested that ALKBH5 was significantly expressed at lower levels in GC tissues, while expression of FTO between GC and normal tissue was not as remarkable as ALKBH5 (Fig. 1H, S1G). Protein level of ALKBH5 in adjacent normal tissues was also higher than the level in GC tissues (Fig. S1I). Hence, we focused on expression of ALKBH5 and its possible mechanism in GC metastasis. Both the TCGA and GEO databases displayed reduced ALKBH5 expression in GC (Fig. 1I-J). Statistical analysis from the tissue microarray also revealed that protein levels of ALKBH5 were significantly higher in adjacent normal tissue (Fig. 1K-L). The receiver operating characteristic (ROC) curves indicated that ALKBH5 was highly sensitive and specific for the clinical diagnosis of GC (Fig. 1M). Survival analysis also indicated that patients with high expression of ALKBH5 exhibited an improved prognosis (Fig. 1N). Clinical statistics revealed that expression of ALKBH5 were lower in patients with distant metastasis than that in patients without distant metastasis (Fig. 1O). Similar results were observed in the lymph node metastasis groups (Fig. 1P). Clinical characterization also revealed that ALKBH5 was closely related to tumor stage and pathological lymph nodes (Fig. S1H, J). Taken together, it was suggested that low expression of ALKBH5 may be the root of GC metastasis.

### ALKBH5 inhibited GC invasion and migration in vitro

**Fig. 2:**
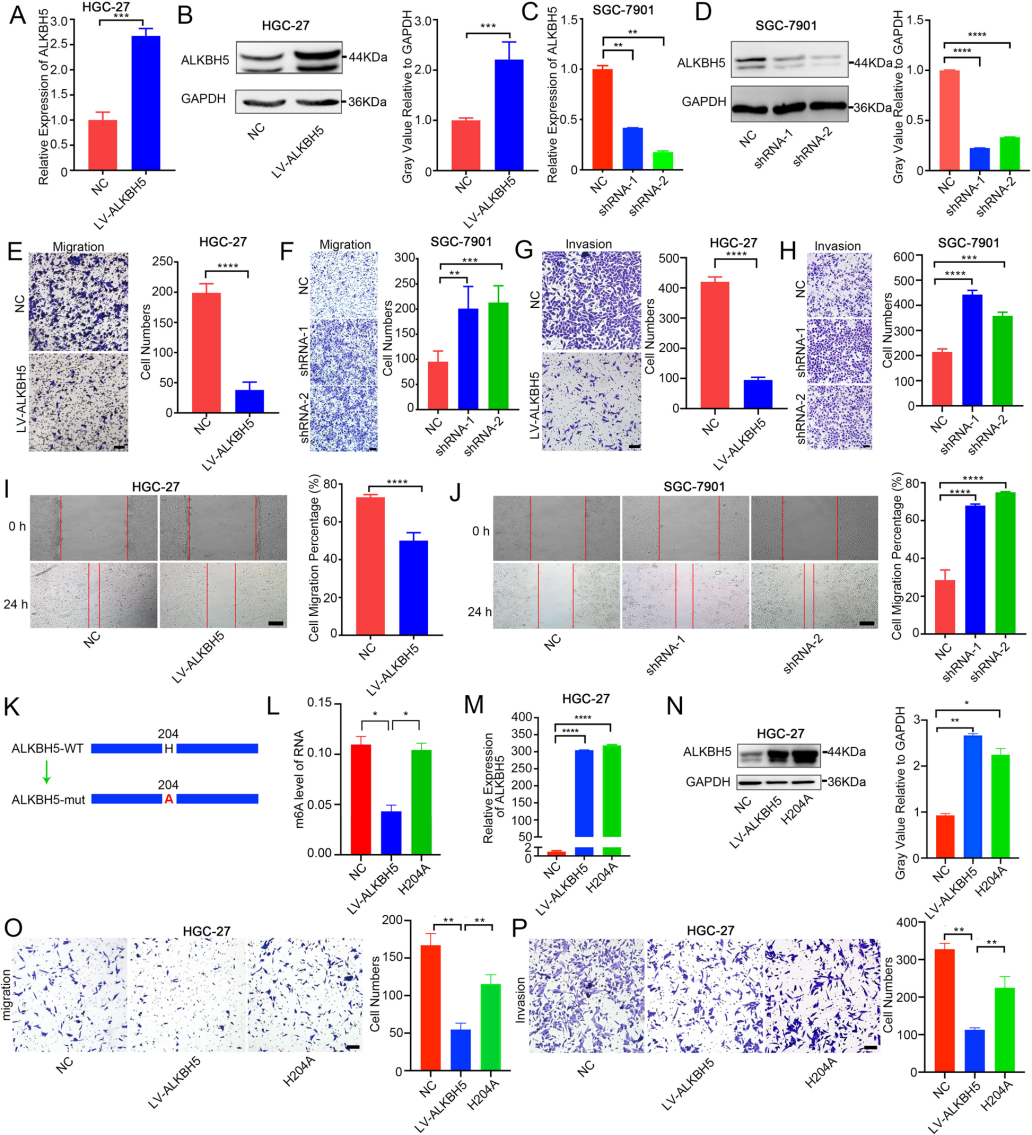
ALKBH5 inhibits cell invasion in vitro experiment (**A**-**B**) MRNA and protein level of ALKBH5 in HGC-27 cell with ALKBH5 overexpression. (**C**-**D**) MRNA and protein level of ALKBH5 in SGC-7901 cell with ALKBH5 knockdown. (**E, G**) Migration and invasion results of overexpressing ALKBH5 in HGC-27 cell, together with its statistical chart (scale bars = 200 µm). (**F,H**) Migration and invasion results of ALKBH5 knockdown in SGC-7901 cell, together with its statistical chart (scale bars = 200 µm). (**I-J**) wound-healing assay in HGC-27 and SGC-7901 cells (scale bars = 200 um). (**K**) Schematic diagram of ALKBH5 H204A. (**L**) M6A level of mRNA in NC, LV-ALKBH5 and ALKBH5 H204A group of HGC-27 cell. (**M–N**) MRNA and protein level of ALKBH5 in LV-ALKBH5 or H204A group of HGC-27 cell. (**O-P**) Migration and invasion results of wild-type ALKBH5 or ALKBH5 H204A in HGC-27 cell, together with its statistical chart (scale bars = 200 µm)

To excavate the potential feature of ALKBH5 in GC progression, we first assessed the expression of ALKBH5 among GC cell lines (Fig. S2A-B). After that, stable overexpression of ALKBH5 was established in HGC-27 and BGC-823 cells (Fig. 2A-B and S2C-D). We also silenced ALKBH5 in SGC-7901 cells (Fig. 2C-D). Validated by transwell assay, the metastatic ability of GC cells were obviously suppressed after ALKBH5 overexpression (Fig. 2E,G and S2E-H), while they were greatly enhanced by ALKBH5 knockdown in SGC-7901 cells (Fig. 2F,H). As expected, Wound-healing assay uncovered that upregulation of ALKBH5 remarkably inhibited migratory ability of GC cells (Fig. 2I, S2I-J). Attenuation of ALKBH5 expression accelerate migration of GC cells (Fig. 2J).

It had been reported that ALKBH5 H204A, which mutates amino acid 204, may result in a deficiency of ALKBH5 demethylase activity [27]. To clarify the role of m6A in migration, we used ALKBH5 H204A mutant in HGC-27 cells (Fig. 2K). Results indicated that mutation of ALKBH5 had less effect on its mRNA or protein levels (Fig. 2M-N), but the m6A level in ALKBH5 H204A cells was obviously higher than that in LV-ALKBH5 GC cells (Fig. 2L). Migration and invasion ability were also enhanced in mutant group when compared with ALKBH5 overexpression (Fig. 2O-P). Rescue experiment was conducted. Wild type or ALKBH5 H204A was overexpressed in ALKBH5-knockdown GC cell (Fig. S2K, N). Transwell assay showed decreased migration and invasion ability after wild-type ALKBH5 overexpression, while H204A had not this inhibitory effect (Fig. S2K-P). These data suggested that the inhibition of cell invasion by ALKBH5 overexpression is mainly dependent on its demethylase activity.

### PKMYT1 was identified as a downstream target of ALKBH5

To explore the potential target of ALKBH5 in GC metastasis, RNA-seq in control and ALKBH5 overexpressing BGC-823 cells was conducted (Fig. S3A). RNA-seq results demonstrated that most transcripts were downregulated in response to ALKBH5 overexpression (Fig.S3A). KEGG and GSEA analysis both showed that genes in which mRNA levels get downregulated were highly enriched in focal adhesion, gap junction and tight junction (Fig. S3B-D). These results implied that the exposure of ALKBH5 can regulate the level of many genes related to invasion and metastasis, suggesting that ALKBH5 is crucial for invasion and metastasis of GC.

**Fig. 3:**
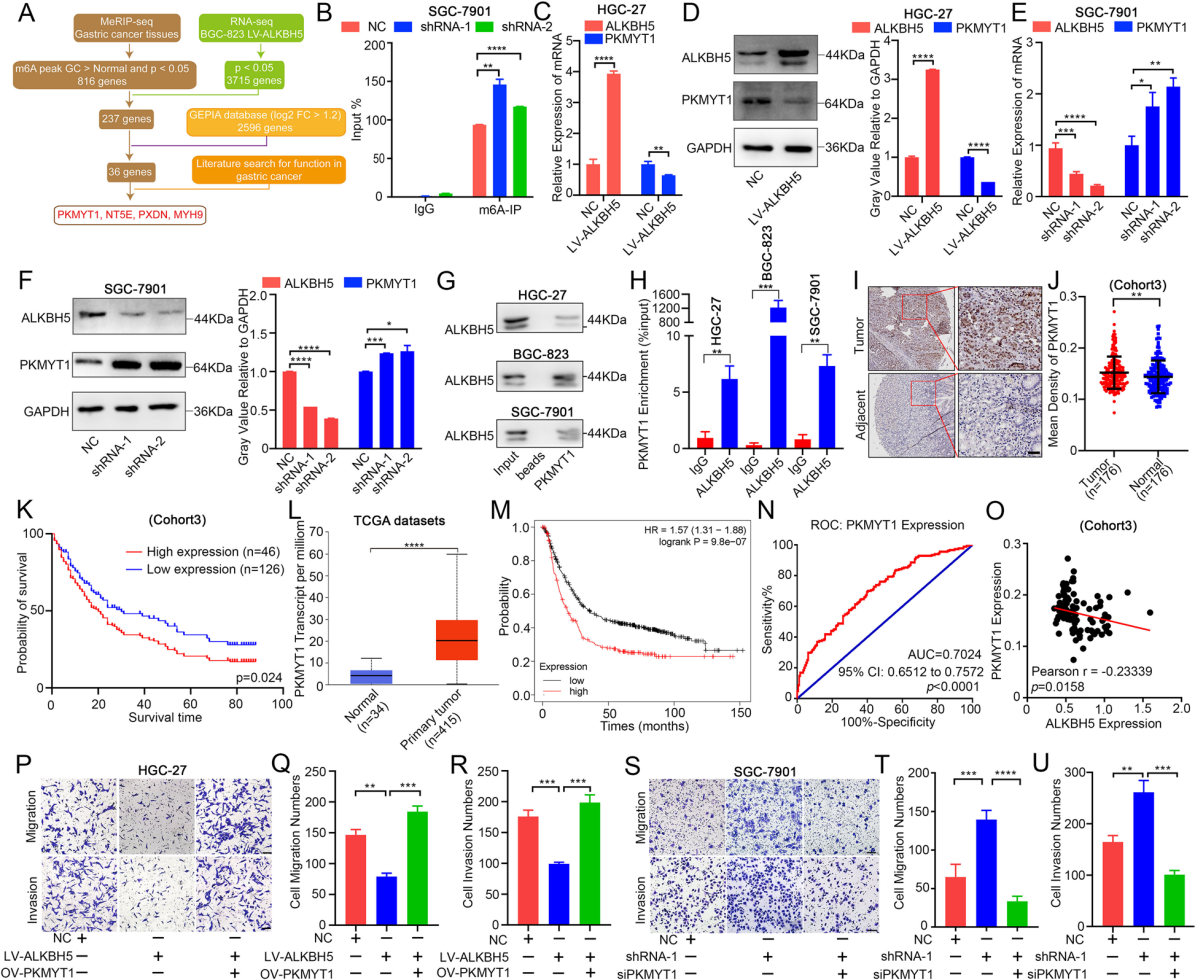
PKMYT1 was identified as a downstream target of ALKBH5 (**A**) Filtering process of target genes among meRIP-seq, mRNA-seq, GEPIA database and literature search. (**B**) MeRIP-qPCR analysis of PKMYT1 after ALKBH5 knockdown in SGC-7901 cell. (**C**-**D**) The protein and mRNA level of PKMYT1 in ALKBH5 overexpression GC cell. (**E**–**F**) The protein and mRNA level of PKMYT1 in ALKBH5 knockdown GC cells. (**G**) RNA Pulldown assay of ALKBH5 using NC or PKMYT1 probe. (**H**) RIP-qPCR assay of PKMYT1 enrichment by ALKBH5 protein. (**I**-**J**) Representative IHC pictures of PKMYT1 in GC tissue microarray (TMA) and the comparison of area density in PKMYT1-staining (scale bars = 100 µm). (**K**) Survival analysis of PKMYT1 expression in GC patients (*p* = 0.024, log-rank test). (**L**) MRNA expression comparison of PKMYT1 in TCGA database. (**M**) Kaplan–Meier OS analysis of PKMYT1 expression in patients with GC (HR = 1.57, *p* = 9.8e-07, log-rank test). (**N**) ROC analysis of PKMYT1 expression in TMA. (**O**) Expression correlation between PKMYT1 and ALKBH5 in TMA. Pearson *r* = -0.23339, *p* = 0.0158. (**P**-**R**) Migration and invasion ability of PKMYT1 overexpression after LV-ALKBH5 in HGC-27 cell (scale bar = 200 um). (**S**-**U**) Migration and invasion ability of si-PKMYT1 after ALKBH5 knockdown in SGC-7901 cell (scale bar = 200 um)

To identify the primary target gene that plays a key downstream of ALKBH5, 816 genes, whose m6A levels were notably higher in GC than that in adjacent tissue, were selected according to the MeRIP-seq result. Combined with 3715 genes that were significantly altered after overexpression of ALKBH5 in the RNA-seq results, we obtained 237 common genes. To further identify the downstream target genes essential in GC, we identified 2596 genes that had significantly high expression (log2 FC > 1.2) in GC using the GEPIA database, and 36 genes were ultimately obtained. A literature review was used to screen four genes (PKMYT1, NT5E, PXDN and MYH9) that have been reported in GC (Fig. 3 A). Using MeRIP-qRT-PCR and mRNA level verification, we found that only PKMYT1 exhibited stable alterations when ALKBH5 was overexpressed or disrupted (Fig. 3B-F, S3E). The m6A levels of PKMYT1 stably increased after ALKBH5 interference in SGC-7901 cells (Fig. 3B). The expression of PKMYT1 were significantly decreased after over-expression of ALKBH5 (Fig. 3C-D) and increased after interference of ALKBH5 (Fig. 3E-F). The other three genes showed inconsistent changes in mRNA levels after overexpression or interference with ALKBH5 (Fig. S3F-G). RIP and RNA pulldown assay both demonstrated the binding between ALKBH5 and PKMYT1 transcript (Fig. 3G-H). Therefore, we preliminarily suspected that PKMYT1 may be a downstream effector of ALKBH5.

Both tissue microarray and TCGA database results indicated highly expressed PKMYT1 in GC, and its expression was strongly correlated with poor prognosis (Fig. 3I-M). ROC analysis suggested significant discrimination of PKMYT1 for clinical GC diagnosis (Fig. 3N). Tissue microarray expression statistics revealed a notably negative correlation between ALKBH5 and PKMYT1 expression (Fig. 3O). To verify the unique role of PKMYT1 in the GC metastasis, we first performed PKMYT1 disruption and overexpression in GC cell lines. The transwell assay demonstrated that the metastatic ability of GC cells was obviously enhanced after PKMYT1 overexpression (Fig. S3H-J). When PKMYT1 was suppressed, the metastatic behavior of GC cells was markedly inhibited (Fig. S3K-M). To observe the effect of ALKBH5/PKMYT1 on GC metastasis, a rescue experiment was carried out. Results showed that overexpression of PKMYT1 significantly restored the metastasis ability caused by ALKBH5 overexpression (Fig. 3P-R), and similar results was observed when disruption of PKMYT1 suppressed the metastasis ability caused by ALKBH5 interference (Fig. 3S-U, S3N-O). These results demonstrates that PKMYT1 functions as a downstream of ALKBH5.

### PKMYT1 promoted invasion and migration of GC in an m6A-dependent manner

**Fig. 4:**
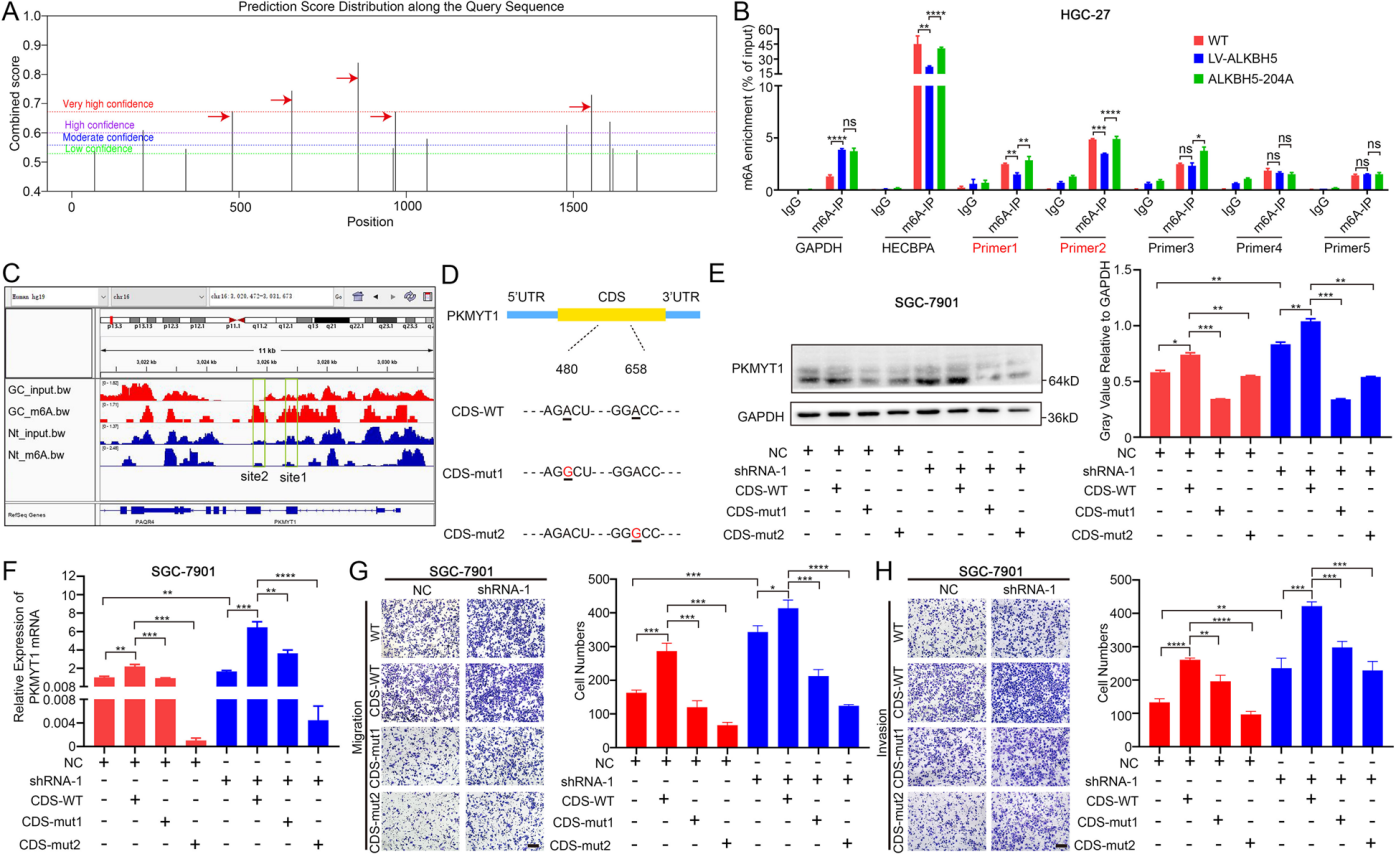
ALKBH5 regulates PKMYT1 via its m6A-dependent manner (**A**) Prediction results of PKMYT1 mRNA in SRAMP website show the potential site of m6A modification. The red arrow points sites with very high confidence. (**B**) MeRIP-qPCR analysis of five sites on PKMYT1 mRNA in HGC-27 cell. GAPDH was regarded as the negative control, while HECBPA belongs to the positive control. (**C**) Peak distribution of the first two sites in MeRIP profiles of GC tissue in IGV genome browser. (**D**) Schematic photo of CDS-WT, CDS-mut1, CDS-mut2 in PKMYT1 mRNA. (**E**–**F**) The protein and mRNA level of PKMYT1 between NC and shRNA-1 group after transfected with CDS-WT, CDS-mut1 and CDS-mut2 in SGC-7901 cell. (**G**-**H**) The migration and invasion ability between NC and shRNA-1 group after transfected with CDS-WT, CDS-mut1 and CDS-mut2 in SGC-7901 cell (scale bar = 200 um)

To identify the specific m6A site affected by ALKBH5, the mRNA sequence of PKMYT1 was predicted using the SRAMP website (http://www.cuilab.cn/sramp/). The prediction results exhibited five potential m6A modification sites with very high confidence in the mRNA sequence of PKMYT1 (Fig. 4A). Then, specific primers for these five sites were designed (Table S1). MeRIP-qRT-PCR indicated that the m6A levels of fragments corresponding to the first two sites were significantly decreased in response to ALKBH5 overexpression (Fig. 4B). When mutating residues involved in the demathylase activity of ALKBH5, their m6A levels get restored (Fig. 4B). The contrary results were observed after ALKBH5 knockdown (Fig. S4A). Our data indicated that the two sites on PKMYT1 mRNA might be the specific position regulated by ALKBH5. The m6A level of these two sites in GC tissues were also checked in the MeRIP-seq. Results from the IGV genome browser demonstrated that the m6A modification level of the two sites were noticeably higher in GC when compared with normal tissue (Fig. 4C). In addition, PKMYT1 were obviously downregulated in the ALKBH5 H204A group (Fig. S4B-C). Mutations in these two sites were designed to observe the effect of m6A modification on PKMYT1 (Fig. 4D). Results revealed that whole level of PKMYT1 was reduced in the mutation group (Fig. 4E-F). The transwell experiments were conducted at the same time. Results showed that mutation of these two sites resulted in a significant reduction in GC metastatic ability (Fig. S4D-F). This phenomenon was more pronounced after ALKBH5 knockdown (Fig. 4G-H, S4G-K). Thus, PKMYT1 promotes invasion and migration of GC in an m6A-dependent manner.

### M6A modification of PKMYT1 mRNA maintained its stability via IGF2BP3

**Fig. 5:**
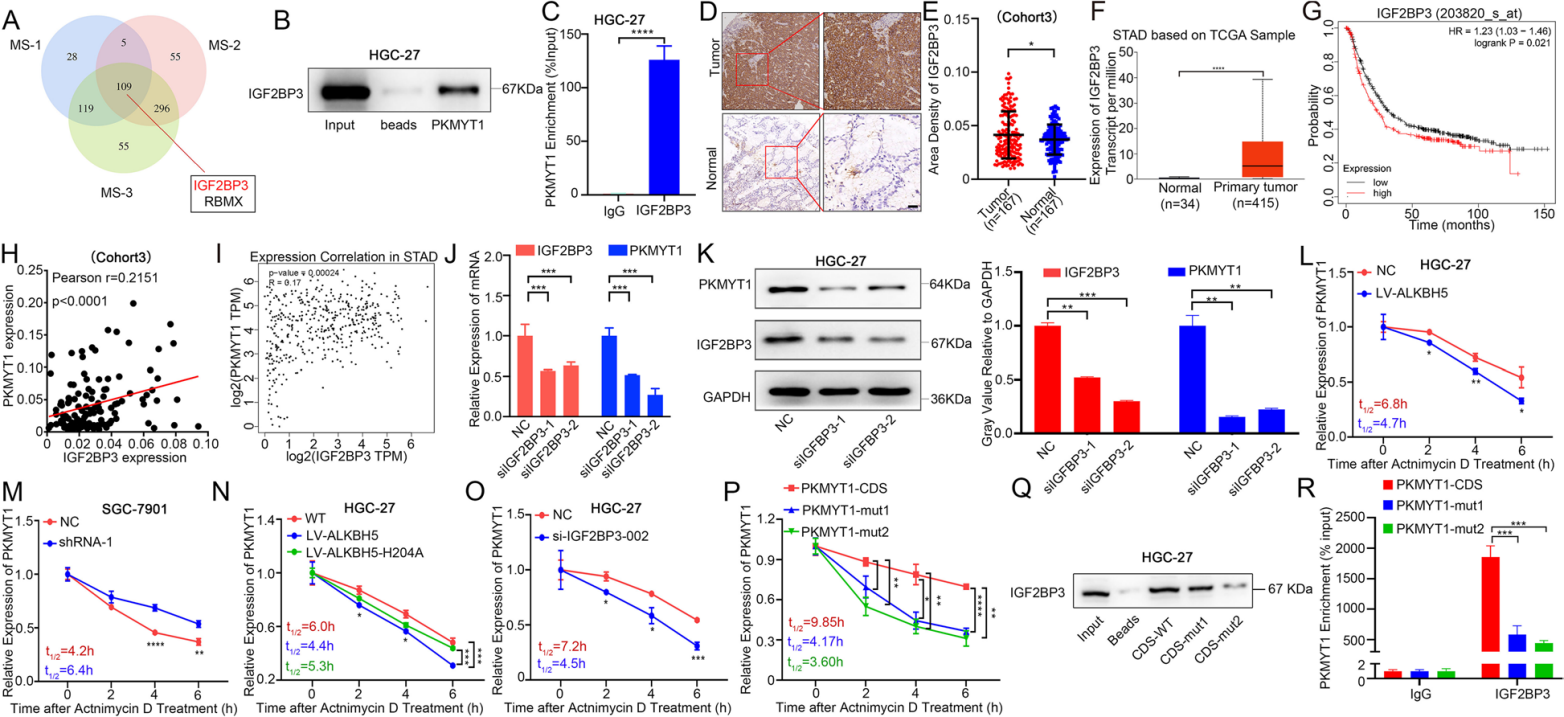
ALKBH5 and IGF2BP3 together regulates the expression of PKMYT1 via its m6A modification (**A**) Results of mass spectrometry using PKMYT1 probe. (**B**) RNA pulldown of endogenous IGF2BP3 using NC or PKMYT1 probe. (**C**) RIP-qPCR assay of PKMYT1 enrichment by IGF2BP3. (**D**-**E**) Representative IHC pictures of IGF2BP3 in GC tissue microarray (TMA) and the comparison of area density in IGF2BP3-staining (scale bars = 100 µm). (**F**) MRNA expression comparison of IGF2BP3 in TCGA database. (**G**) Kaplan–Meier OS analysis of IGF2BP3 expression in patients with GC (HR = 1.23, *p* = 0.021, log-rank test) (**H**) Correlation analysis of PKMYT1 and IGF2BP3 expression in TMA. Pearson *r* = 0.2151, *p* < 0.0001. (**I**) Expression correlation between PKMYT1 and IGF2BP3 in STAD of TCGA database. Pearson *r* = 0.17, *p* = 0.00024. (**J**-**K**) The mRNA and protein level of PKMYT1 in si-IGF2BP3 HGC-27 cell. (**L**-**N**) RNA stability of PKMYT1 mRNA in ALKBH5-overexpressing, ALKBH5 knockout and H204A GC cells after treated with actinomycin D (5 µg/mL). (**O**) MRNA level of PKMYT1 at the indicated time points after actinomycin D treatment in si-IGF2BP3 HGC-27 cell. (**P**) RNA stability of PKMYT1 mRNA in PKMYT1-CDS, CDS-mut1 and CDS-mut2 groups. (**Q**-**R**) RNA-pulldown and RIP assay between PKMYT1 mRNA and IGF2BP3 after mutation in PKMYT1-CDS

It is well known that m6A modification primarily depends on the “reader” protein to exert additional function [28]. To further investigate the potential “reader” protein in m6A modification of PKMYT1, RNA pulldown experiment together with mass spectrometry assay were carried out. The results of mass spectrometry showed that IGF2BP3 and RBMX may play a role after m6A modification of PKMYT1, while the score of IGF2BP3 (score = 69.33) was much higher than the score of RBMX (score = 29.05) (Fig. 5 A). We chose IGF2BP3 for validation. RNA pulldown and RIP assays both indicated that IGF2BP3 could bind with PKMYT1 mRNA (Fig. 5B-C, and S5A-D). Tissue microarray and TCGA database both showed highly expressed IGF2BP3 in GC and its high expression conveyed poor prognosis to GC patients (Fig. 5D-G). Both tissue microarray and TCGA database results showed a remarkable positive correlation between IGF2BP3 and PKMYT1 expression in GC (Fig. 5H-I). Validation experiments in cell lines confirmed that expression of PKMYT1 was remarkably decreased after interfering with IGF2BP3 (Fig. 5J-K).

It has been reported that IGF2BP3, an m6A reader protein, primarily functions by enhancing mRNA stability [29, 30]. Therefore, the stability of PKMYT1 mRNA after interfering with and overexpressing ALKBH5 was assessed. It was found that the mRNA stability of PKMYT1 was significantly decreased after overexpression of ALKBH5 under actinomycin D treatment but increased distinctly after interference with ALKBH5 (Fig. 5L-M). In the ALKBH5 H204A group, the stability of PKMYT1 mRNA was restored (Fig. 5N). A notable reduction in the mRNA stability of PKMYT1 was observed after interfering with the reading protein IGF2BP3 (Fig. 5O). To investigate the influence of m6A sites modification to PKMYT1 mRNA stability, GC cells were transfected with PKMYT1-CDS, mut1, and mut2 plasmids under treatment with actinomycin D. It was observed that mRNA stability of PKMYT1 was also reduced after these two sites were mutated (Fig. 5 P).

To further explore the relationship between IGF2BP3 and m6A modification sites of PKMYT1, in vitro transcription assay together with biotin labeling was performed to synthesize full length mRNA sequences containing the single m6A modification site mutation in PKMYT1, respectively. In RNA pulldown assay, streptavidin-conjugate dynabeads were used to verify the direct interaction between IGF2BP3 and PKMYT1 mRNA. It was found that the binding ability of IGF2BP3 to PKMYT1 mRNA was significantly declined after the mutation of these two sites (Fig. 5Q). Similar result was confirmed by RIP assay (Fig. 5 R).

### Correlation between ALKBH5, PKMYT1 and IGF2BP3 in vivo

**Fig. 6:**
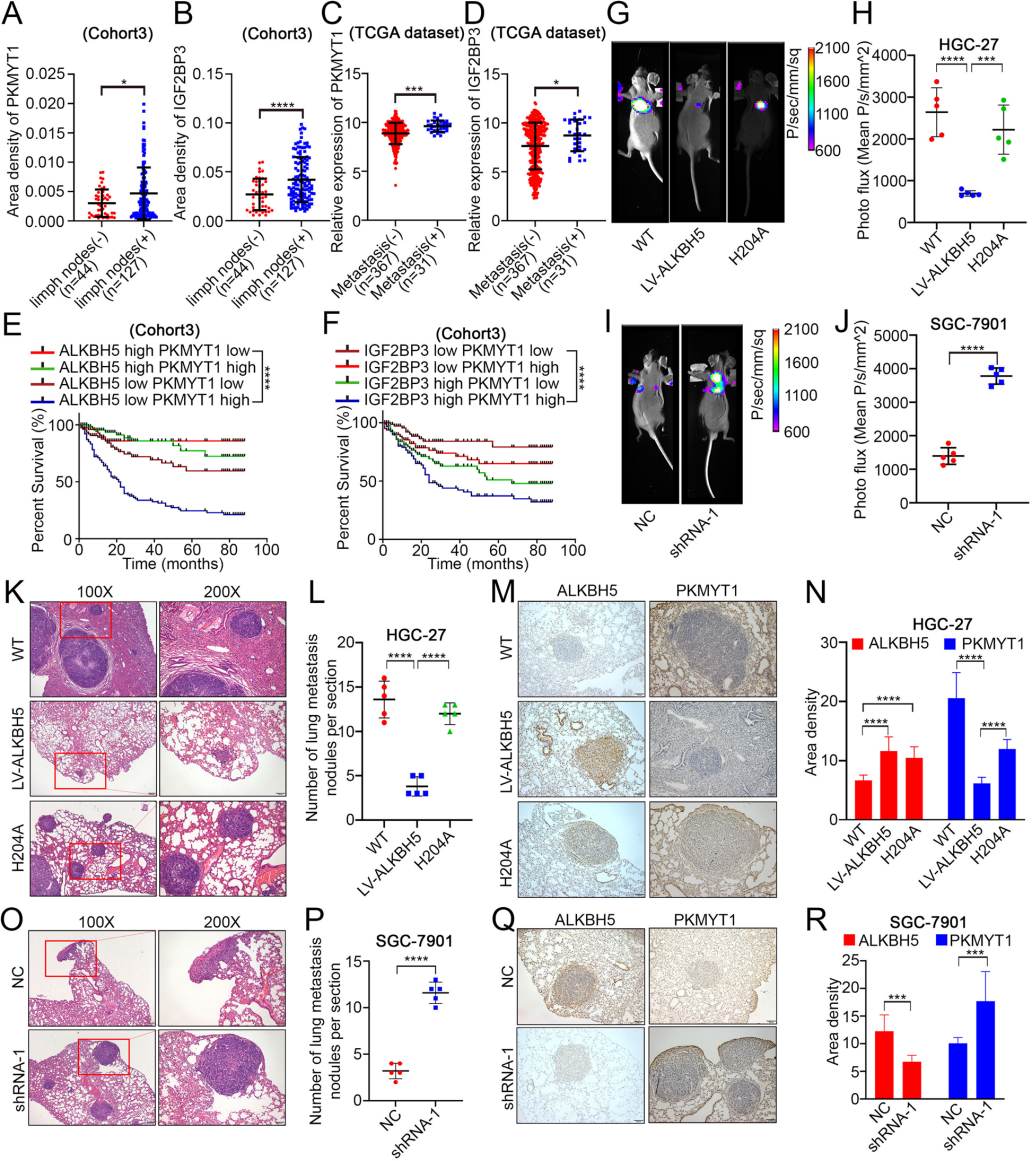
ALKBH5-PKMYT1-IGF2BP3 axis inhibits invasion and migration in vivo experiment (**A**-**D**) Expression comparison of PKMYT1 and IGF2BP3 between metastasis-/ + and limph nodes metastasis-/ + group in TCGA dataset and TMA. (**E**–**F**) Survival analysis of ALKBH5, PKMYT1 expression and PKMYT1, IGF2BP3 expression group in TMA. (**G**-**H**) Representative live images and lung fluorescence statistics after tail vein injection with WT, LV-ALKBH5 and H204A GC cells. (**I**-**J**) Representative live images and lung fluorescence statistics after tail vein injection with NC and shRNA-1 GC cells. (**K**-**L**, **O**-**P**) HE staining of lung sections and statistical analysis on number of lung tumor nodules. (**M**–**N**, **Q**-**R**) Representative IHC pictures of ALKBH5 and PKMYT1 and their area density in lung sections

Analysis on the expression of PKMYT1 and IGF2BP3 in the tumor distant metastasis group and lymph node metastasis group was performed using a tissue microarray and TCGA database, respectively (Fig. 6A-D). Expression of PKMYT1 in metastasis group was obviously higher than that in non-metastasis group (Fig. 6A,C). IGF2BP3 showed the same phenomenon as well as PKMYT1 (Fig. 6B,D). Survival analysis displayed that patient with high-ALKBH5 and low-PKMYT1 expression showed the best prognosis (Fig. 6 E).

Notably, patients with low expression of both PKMYT1 and IGF2BP3 also showed the best prognosis (Fig. 6F). Interestingly, the expression of ALKBH5, PKMYT1 and IGF2BP3 in digestive tract tumors exhibited a similar expression tendency, according to the TCGA database (Fig. S5E). This suggested that the ALKBH5/PKMYT1/IGF2BP3 regulation system might play an important role in digestive tract tumors.

A tail vein injection method was next used to establish a model of tumor lung metastasis in nude mice. Formation of pulmonary metastasis was observed via Molecular Imaging Software. Overexpression of ALKBH5 reduced the ability of these cells to form lung metastasis (Fig. 6G-H), while mutation of ALKBH5 (H204A) rescued this ability (Fig. 6G-H). Oppositely, knockdown of ALKBH5 substantially accelerated the formation of metastasis in mice (Fig. 6I-J). HE staining of lung tissue sections also revealed a strikingly reduced numbers of metastatic nodules in response to ALKBH5 overexpression in GC cells (Fig. 6K-L). IHC was conducted to evaluate the correlation of ALKBH5 and PKMYT1 expression in metastatic nodules of lung tissue slices (Fig. 6M-N). Expression of PKMYT1 was obviously inhibited after ALKBH5 overexpression but get recovered after mutation of ALKBH5 (Fig. 6M-N). The contrary results were observed after ALKBH5 knockdown (Fig. 6O-R).

## Discussion

As one of the most distinctive features of malignancy, invasion and metastasis are the leading cause of fatality in GC patients [31–34]. Mechanisms related to RNA modification and metastasis have been published in gastric cancer [17, 35, 36]. A better comprehension of RNA modification in GC invasion and metastasis is vital for the evolution of new innovative curative strategies [37, 38].

Among RNA modifications, m6A is the most prevalent type in mRNA and noncoding RNA [39]. As a dynamic regulation process, “writers” and “erasers” together control the balance of m6A level in human body [40]. Growing evidence suggests that m6A regulators play an instrumental role in various physiological and pathological diseases by regulating the epigenetic transcriptional levels of genes [41]. Human fetal tissue sequencing has also revealed a tight correlation between m6A modification and gene expression homeostasis [22]. M6A modification is involved in different types of human diseases, such as cardiovascular disease [42], chronic obstructive pulmonary disease [43], liver carcinogenesis [44] and colorectal cancer [45]. Nevertheless, the function of m6A in regulating invasion and metastasis of GC remains largely elusive.

ALKBH5, an important participant in m6A methylation modification, has also been reported in other tumors, such as leukemia [46], glioblastoma [47], pancreatic cancer [48], lung cancer [49] and breast cancer [50]. Recent findings have also highlighted the controversial role of m6A in cancer progression. For instance, ALKBH5 was reported to show highly expression in acute myeloid leukemia (AML) and to regulate the stability of AXL mRNA to maintain leukemia stem cell (LSC) function [51], nonetheless, recent reports have shown that ALKBH5 plays an inhibitory role in pancreatic cancer [52]. It is speculated that ALKBH5 may play a role in regulating different target genes or m6A modifications in different regions of the same gene, or due to the role of different reading proteins.

In this study, we first reported that the major m6A “eraser”, ALKBH5, had an essential effect on GC invasive metastasis. We exhibited the downregulation of ALKBH5 and its clinical significance, predicting its underlying value in GC prognosis. M6A-seq and mRNA-seq were used to quantitatively compare changes in tumors and adjacent normal tissues at the epigenetic and transcriptional level. The results showed that most genes highly modified by m6A also exhibited high mRNA expression levels in GC. Here, we found that ALKBH5 may represent a key regulator of tumor metastasis, and its overexpression significant inhibited tumor metastasis. Statistical analysis of clinical characteristics displayed that elevated expression of ALKBH5 was positively associated with a good prognosis and suppressed distant tumor metastasis and lymph node metastasis in patients. It has been reported that METTL3 can be considered as a clinical prognostic indicator and significantly promotes metastasis in GC [17]. We speculate that ALKBH5 may act in the opposite direction by regulating key target genes.

PKMYT1, a partner of the serine/threonine protein kinase family, was considered as a poor prognosis marker among many different types of tumors, such as glioblastoma [53], ovarian cancer [54], prostate cancer [55], and esophageal squamous cell carcinoma [56]. Reports have shown that PKMYT1 activates the notch signaling pathway and enhances proliferation and tumorigenesis in lung adenocarcinoma [57, 58]. PKMYT1 can also be inhibited by the upstream MCSR1, thus inhibiting GC cell invasion [59]. Beta-catenin/TCF signaling can be activated by PKMYT1 to promote cell invasion and migration in hepatocellular carcinoma [60]. However, there is no distinct report on the reasons related to the upregulation of PKMYT1 expression in GC. We found that ALKBH5 could act as an upstream regulator of PKMYT1, which could be mediated by ALKBH5 and thus remove m6A modification and up-regulate its expression.

**Fig. 7:**
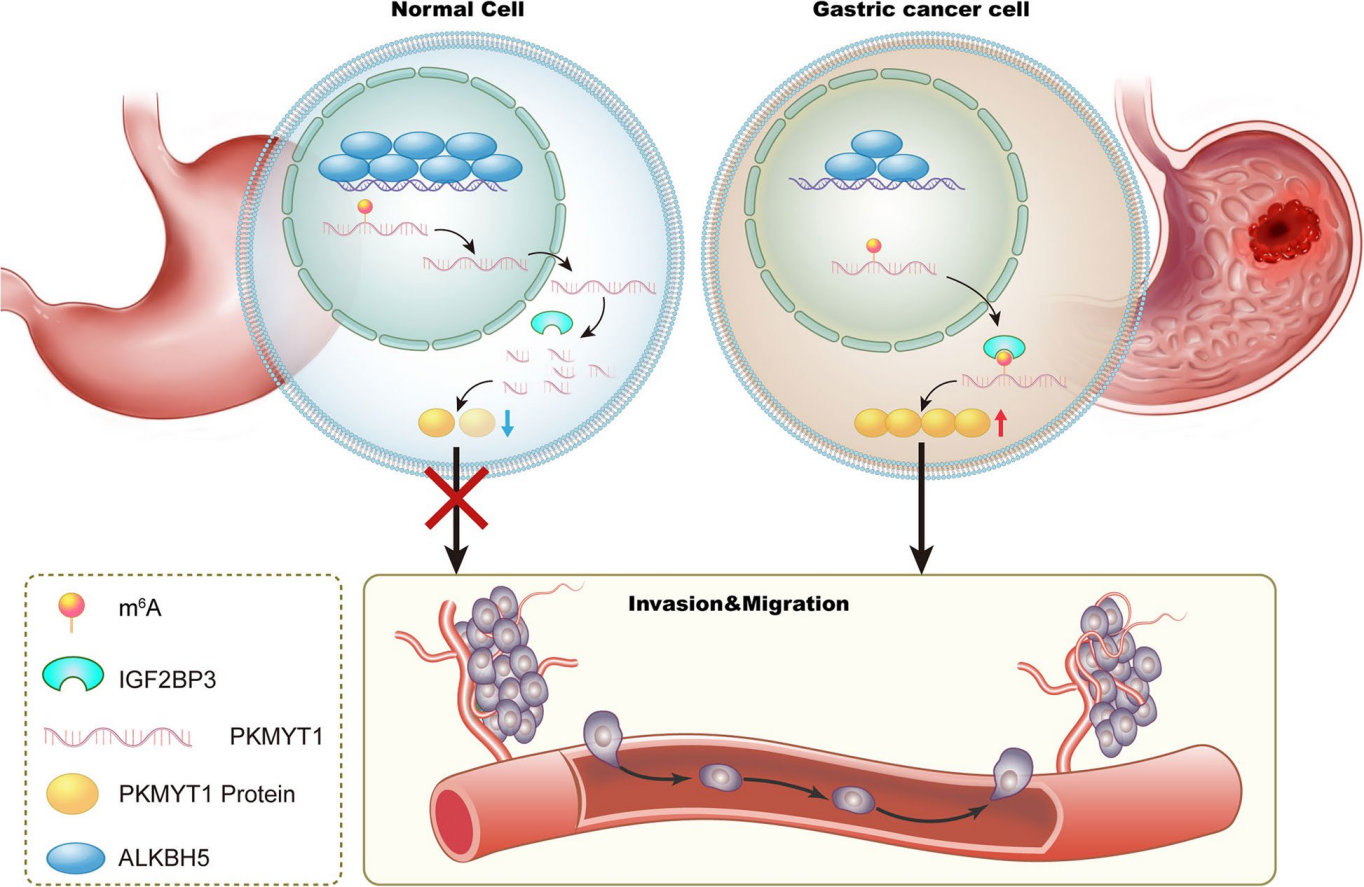
graphic illustration

It has been generally recognized that an intermediate bridge between the alteration of m6A modification level of target genes and their expression level is required by m6A “readers”. And the “readers” that are currently reported to play a role in GC include HuR (also known as ELAVL1) [17], IGF2BP1 [61] and IGF2BP3 [18]. IGF2BP3 regulates metastasis of melanoma [62] and GC cells [63]. Here, it was found that "reader" protein IGF2BP3 regulated the mRNA and protein levels of PKMYT1 by enhancing its mRNA stability, and mutation of modification site in PKMYT1 could decrease its binding with IGF2BP3 (Fig. 7).

Both the literature and our findings indicate that m6A modification is crucial for the metastasis of GC, therefore, targeting dysfunction of m6A regulators represents a promising strategy for cancer therapy. Several studies have shown that small molecule inhibitors targeting dysfunction of m6A regulators have therapeutic potential for cancer [15]. Furthermore, since m6A modifications also play a vital role in mediating cancer responses to chemotherapy, radiotherapy and immunotherapy, targeting m6A regulators could also be applied clinically along with chemotherapy, radiotherapy, or immunotherapy to achieve improved cancer therapeutics in the near future. In addition, mutation or dysregulation of functionally essential m6A sites of m6A regulators can also be targeted, and such manipulation may also be applied in future cancer treatment clinics. Overall, the study of m6A modifications in cancer represents a new frontier in cancer research, revealing a new level of epigenetic regulation in cancer and providing new insights into the molecular mechanisms of tumor development.

## Conclusions

Overall, we have identified ALKBH5 as a tumor suppressor in GC metastasis, and this role is dependent on the demethylase activity of ALKBH5. PKMYT1, a downstream target gene of ALKBH5, can be recognized and bound by the “reader” protein IGF2BP3 after m6A modification. The mRNA stability of PKMYT1 gets enhanced, resulting in higher expression level and ultimately a significant promotion on GC metastasis.

## Supplementary Information

**Additional file 1: Figure S1.** ALKBH5 was correlated with tumor stage and lymph node metastasis in database analysis.

**Additional file 2: Figure S2.** ALKBH5 inhibited invasion and migration in BGC-823 cell.

**Additional file 3: Figure S3.** PKMYT1 promoted invasion and migration in GC cell.

**Additional file 4: Figure S4.** Mutation of m6A modification site suppressed invasion and migration ability of PKMYT1.

**Additional file 5: Figure S5.** ALKBH5/PKMYT1/IGF2BP3 regulation system may exist among digestive system.

**Additional file 6: Table S1.** RT-PCR Primer Sequences.

**Additional file 7.** The specific sequence of CDS and mut plasmids in PKMYT1.

**Additional file 8.** PKMYT1 mRNA-SRAMP prediction Results.

**Additional file 9.** 823-RNA-seq Gene_differential_expression.

**Additional file 10.** 823-RNA-seq GSEA gene list.

## Data Availability

All data analyzed during this study are included in this published article and its supplementary information files.

## Abbreviations

GC: Gastric cancer
m6A: N6-methyladenosine
ALKBH5: AlkB homolog 5
MeRIP-seq: Methylated RNA immunoprecipitation sequencing
qRT-PCR: Quantitative real-time PCR
IHC: Immunohistochemistry
RIP: RNA immunoprecipitation
METTL3: Methyltransferase-like 3
ZMYM1: Zinc finger MYM-type containing 1
BCA: Bicinchoninic acid
siRNAs: Small interfering RNAs
H&E: Hematoxylin–eosin
GO: Gene ontology
GSEA: Gene set enrichment analysis
TMA: Tissue microarray
IOD: Integrated optical density
ROC: Receiver operating characteristic
LSC: Leukemia stem cell
