# Supplementary material for: Demethylase ALKBH5 suppresses invasion of gastric cancer via PKMYT1 m6A modification
Source: Mol Cancer. 2022 Feb 3;21:34. doi: 10.1186/s12943-022-01522-y (PMC8812266; doi:10.1186/s12943-022-01522-y)
Supplement: Supplementary file 1 — Additional file 1: Figure S1. ALKBH5 was correlated with tumor stage and lymph node metastasis in database analysis. [file 12943_2022_1522_MOESM1_ESM.docx]

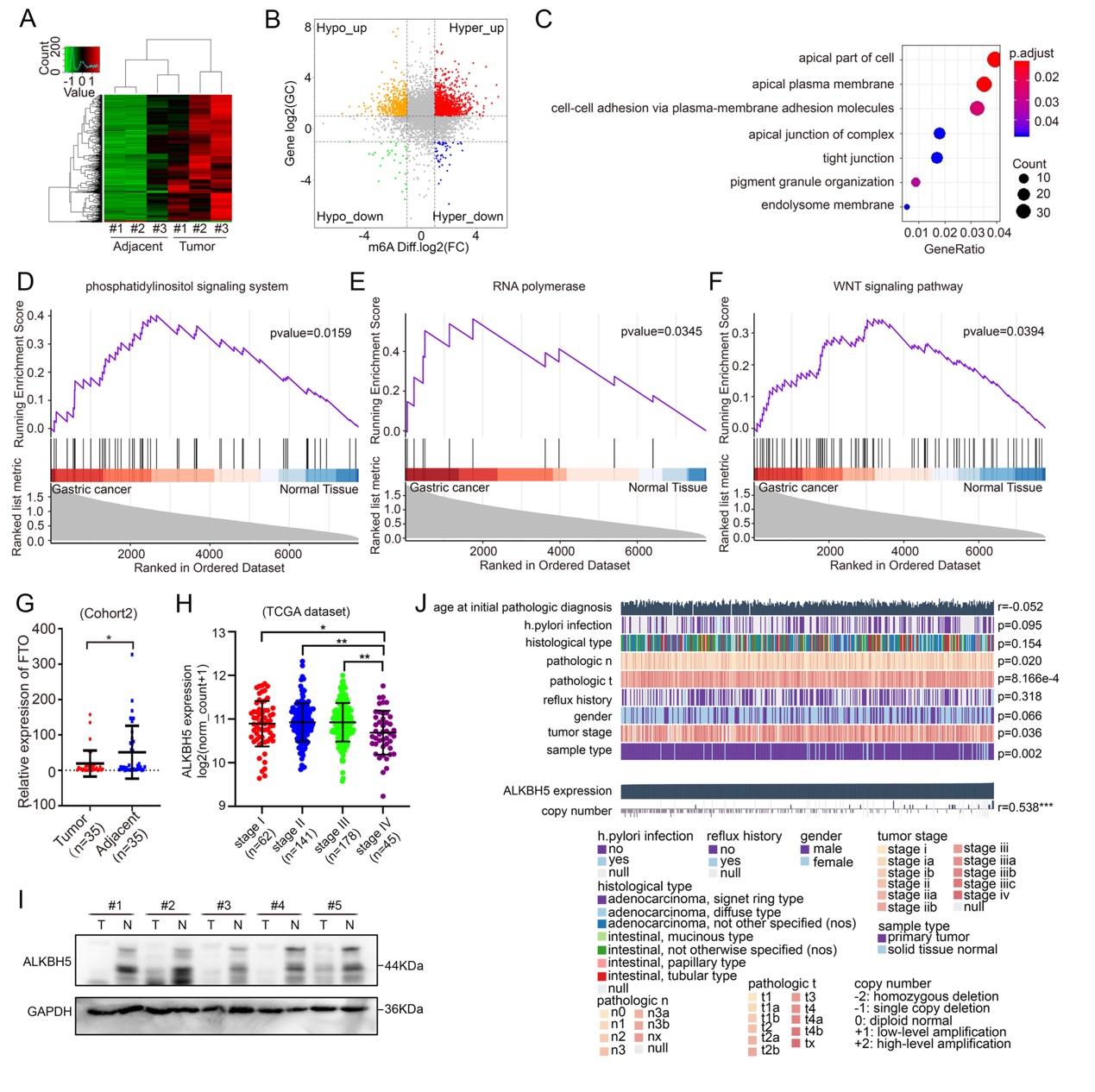


Figure S1. ALKBH5 was correlated with tumor stage and lymph node metastasis in database analysis

(A) Heat map of genes in meRIP-seq results shows mRNA level in tumor tissues.

(B) Quadrant Chart of m6A and mRNA fold change of genes in MeRIP-seq data.

(C) KEGG analysis showed tight juncation and endosomal membrane enrichment in the sequencing profile.

(D-F) Gene set enrichment analysis (GSEA) indicated significant enrichment in phosphatidylinositol signaling system, RNA polymerase and WNT signaling pathway

(G) MRNA expression comparison of FTO between tumor and normal tissues in cohort2 (n=35).

(H) ALKBH5 expression among different pathologic stage of STAD in TCGA database.

(I) Protein level of ALKBH5 in 5 pairs of clinical tumor samples.

(J) Analysis of TCGA patient tumor data showing mRNA expression pattern of ALKBH5 across stages of GC patient samples.
