# Supplementary material for: Demethylase ALKBH5 suppresses invasion of gastric cancer via PKMYT1 m6A modification
Source: Mol Cancer. 2022 Feb 3;21:34. doi: 10.1186/s12943-022-01522-y (PMC8812266; doi:10.1186/s12943-022-01522-y)
Supplement: Supplementary file 2 — Additional file 2: Figure S2. ALKBH5 inhibited invasion and migration in BGC-823 cell. [file 12943_2022_1522_MOESM2_ESM.docx]

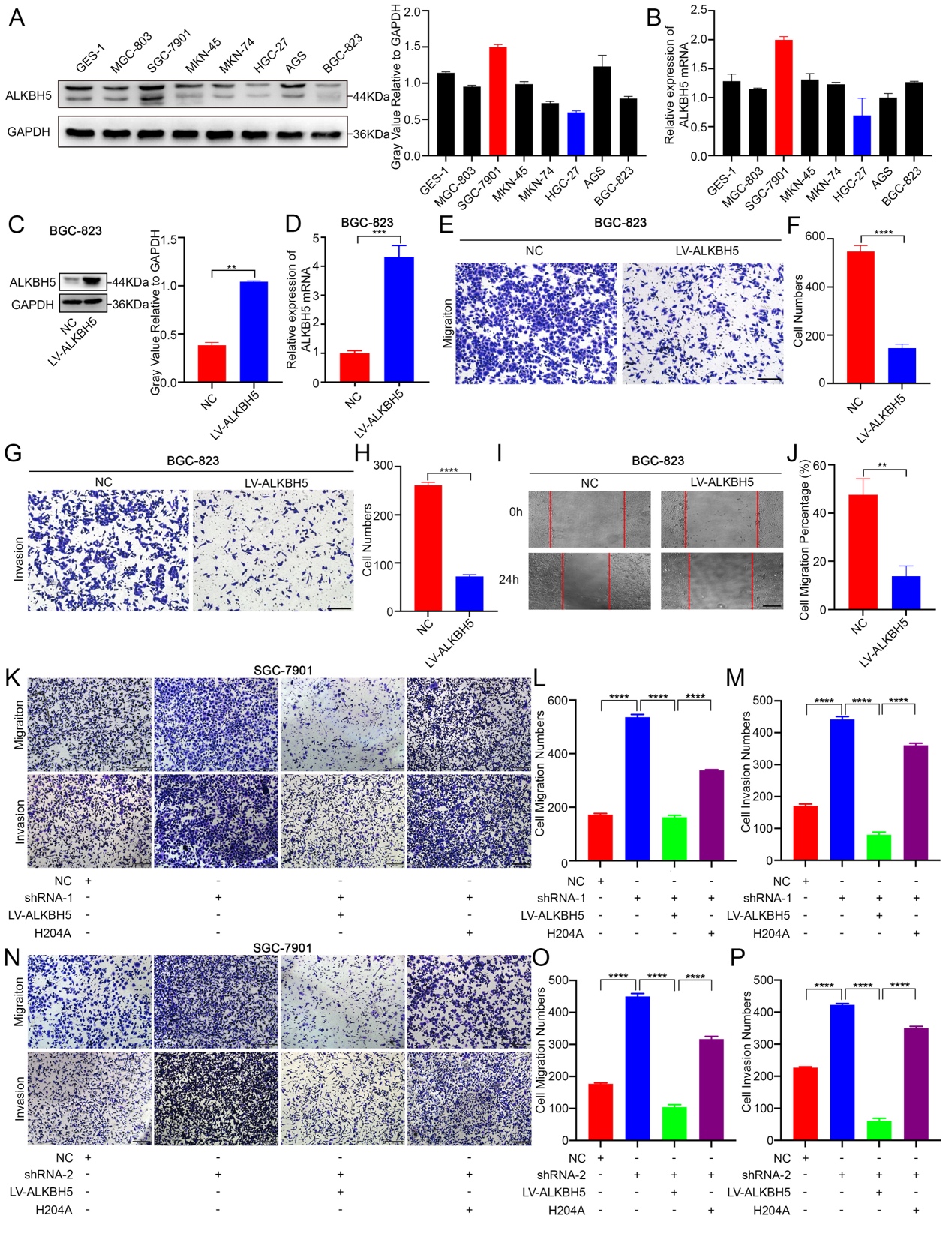


Figure S2. ALKBH5 inhibited invasion and migration in BGC-823 cell.

(A-B) The protein and mRNA level of ALKBH5 among GC cell lines.

(C-D) The protein and mRNA level of ALKBH5 in BGC-823 cell with ALKBH5 overexpression.

(E-H) Migration and invasion of ALKBH5 overexpression in BGC-823 cell (scale bars=200 µm).

(I-J) Wound-healing assay in BGC-823 cell after ALKBH5 overexpression (scale bars=200 um).

(K) Transwell assay of overexpressing wild-type ALKBH5 or H204A in shRNA-1 SGC-7901 GC cell (scale bars=200 µm).

(L-M) Migration and invasion cell numbers in figure S2K.

(N) Transwell assay of overexpressing wild-type ALKBH5 or H204A in shRNA-2 SGC-7901 GC cell (scale bars=200 µm).

(O-P) Migration and invasion cell numbers in figure S2N.
