# Supplementary material for: Demethylase ALKBH5 suppresses invasion of gastric cancer via PKMYT1 m6A modification
Source: Mol Cancer. 2022 Feb 3;21:34. doi: 10.1186/s12943-022-01522-y (PMC8812266; doi:10.1186/s12943-022-01522-y)
Supplement: Supplementary file 3 — Additional file 3: Figure S3. PKMYT1 promoted invasion and migration in GC cell. [file 12943_2022_1522_MOESM3_ESM.docx]

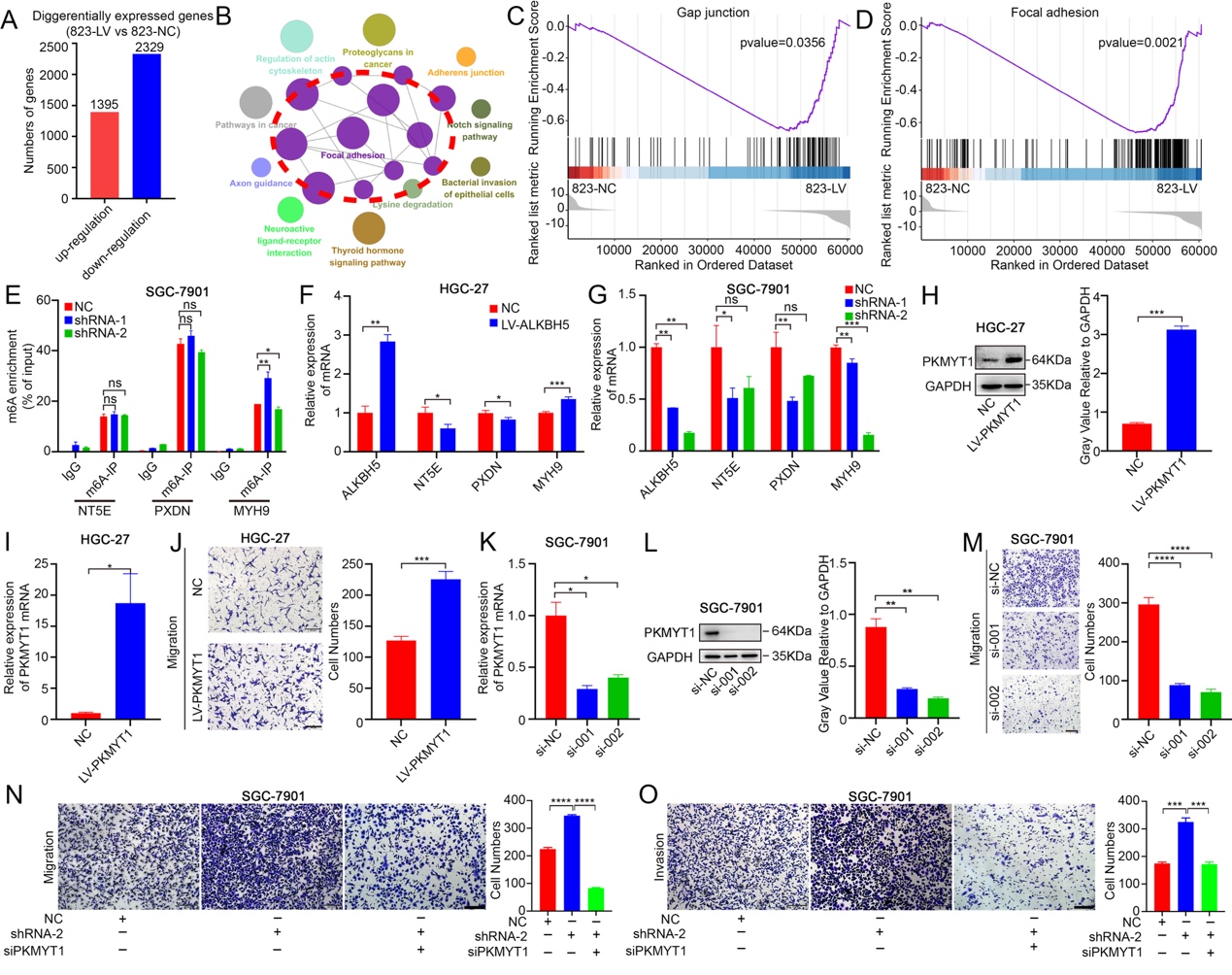


Figure S3 RNA-seq indicated relationship between ALKBH5 and cell adhesion, and PKMYT1 promoted invasion and migration in GC cell.

(A) Number of genes which mRNA expression get altered after ALKBH5 overexpression in BGC-823 cell.

(B-D) KEGG and GSEA analysis both show enrichment of focal adhesion and gap junction after ALKBH5 overexpression.

(E) MeRIP-qPCR analysis of NT5E, PXDN and MYH9 after ALKBH5 knockdown in SGC-7901 cell.

(F-G) The mRNA level of NT5E, PXDN and MYH9 in ALKBH5 overexpression and knockdown GC cells were measured by qRT-PCR.

(H-I) The protein and mRNA level of PKMYT1 were validated by western blotting and qRT-PCR after overexpression of PKMYT1.

(J) The migration assay of GC cell after PKMYT1 overexpression.

(K-L) The protein and mRNA level of PKMYT1 in si-PKMYT1 SGC-7901 cell.

(M) Transwell assay of GC cell after si-PKMYT1.

(N-O) Migration and invasion assay of siPKMYT1 in shRNA-2 SGC-7901 GC cell.
