# Supplementary material for: Demethylase ALKBH5 suppresses invasion of gastric cancer via PKMYT1 m6A modification
Source: Mol Cancer. 2022 Feb 3;21:34. doi: 10.1186/s12943-022-01522-y (PMC8812266; doi:10.1186/s12943-022-01522-y)
Supplement: Supplementary file 4 — Additional file 4: Figure S4. Mutation of m6A modification site suppressed invasion and migration ability of PKMYT1. [file 12943_2022_1522_MOESM4_ESM.docx]

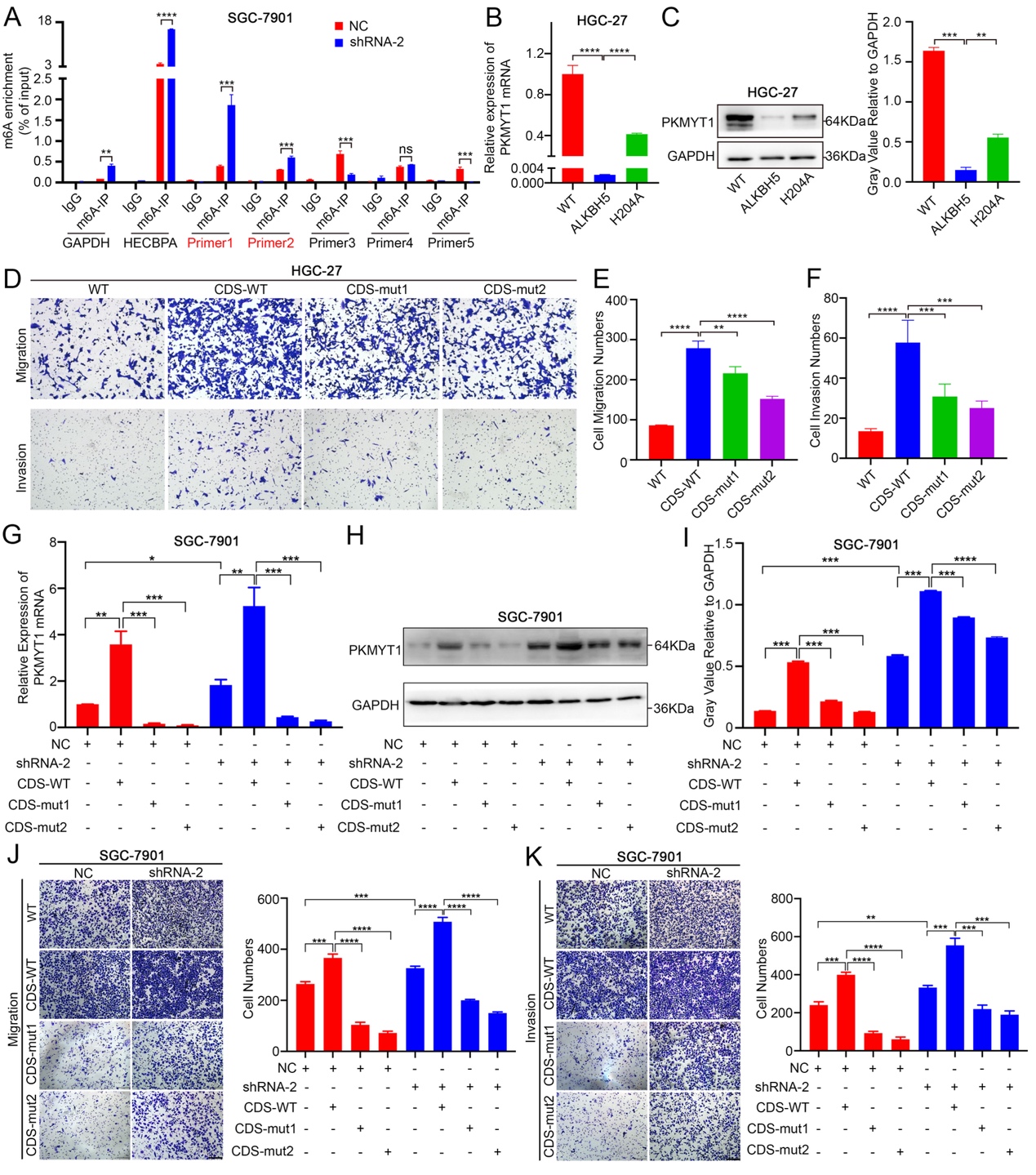


Figure S4. Mutation of m6A modification site suppressed invasion and migration ability of PKMYT1.

(A) MeRIP-qPCR analysis of five sites on PKMYT1 mRNA after ALKBH5 knockdown in SGC-7901 cell.

(B-C) MRNA and protein level of PKMYT1 in LV-ALKBH5 and H204A HGC-27 cell.

(D-F) The migration and invasion ability of HGC-27 GC cell after transfected with plasmids of CDS region mutation in PKMYT1.

(G-I) The protein and mRNA level of PKMYT1 between NC and shRNA-2 group after transfected with CDS-WT, CDS-mut1 and CDS-mut2 in SGC-7901 cell.

(J-K) The migration and invasion ability between NC and shRNA-2 group after transfected with CDS-WT, CDS-mut1 and CDS-mut2 in SGC-7901 cell (scale bar=200 um).
