# Supplementary material for: Demethylase ALKBH5 suppresses invasion of gastric cancer via PKMYT1 m6A modification
Source: Mol Cancer. 2022 Feb 3;21:34. doi: 10.1186/s12943-022-01522-y (PMC8812266; doi:10.1186/s12943-022-01522-y)
Supplement: Supplementary file 5 — Additional file 5: Figure S5. ALKBH5/PKMYT1/IGF2BP3 regulation system may exist among digestive system. [file 12943_2022_1522_MOESM5_ESM.docx]

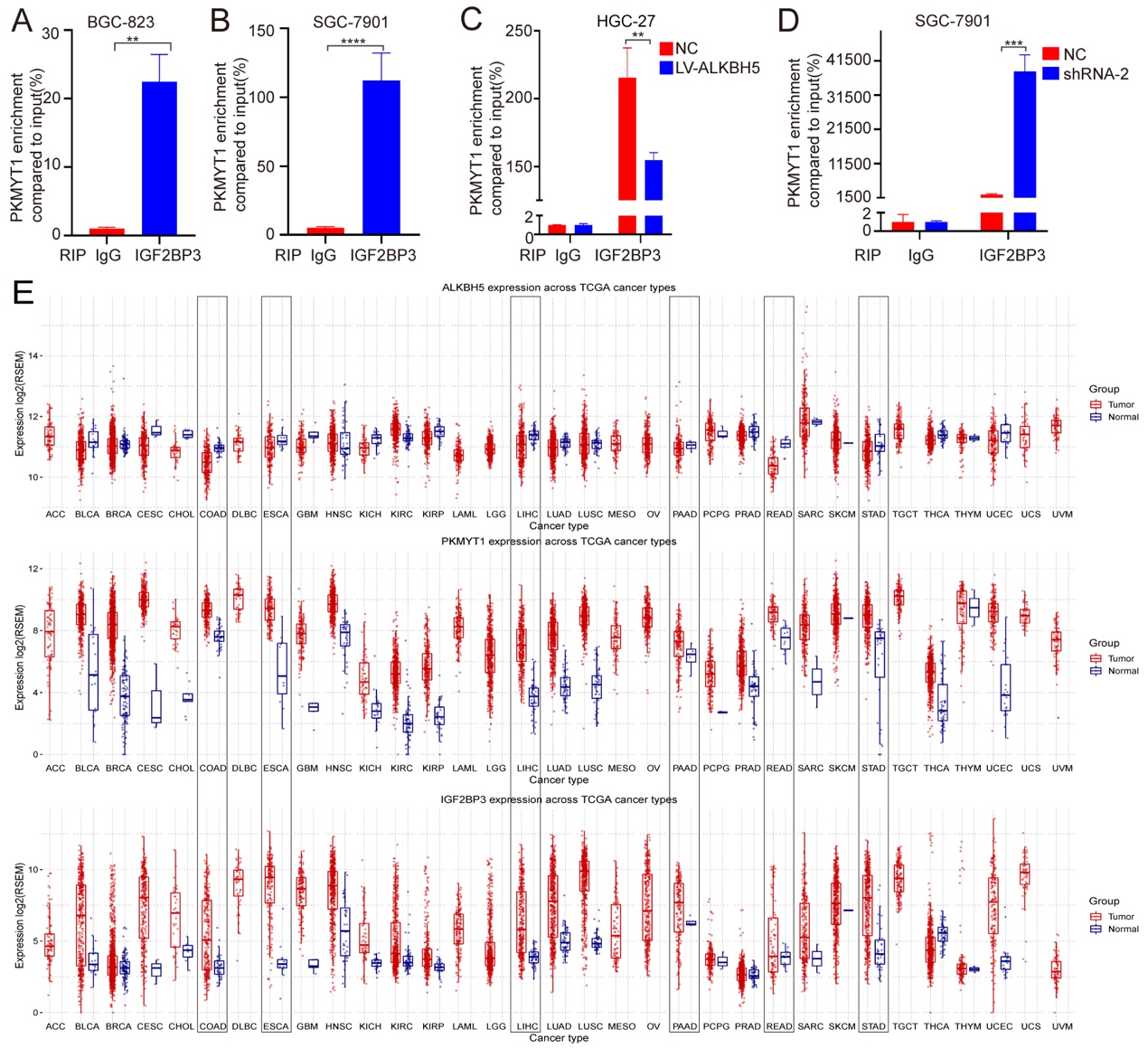


Figure S5. ALKBH5/PKMYT1/IGF2BP3 regulation system may exist among digestive system.

(A-B) RIP-qPCR assay of PKMYT1 enrichment by IGF2BP3 in BGC-823 and SGC-7901 cell.

(C) The enrichment of PKMYT1 in ALKBH5-overexpressing HGC-27 GC cell.

(D) The enrichment of PKMYT1 in ALKBH5-knockdown SGC-7901 GC cell.

(E) Expression of ALKBH5, PKMYT1 and IGF2BP3 among digestive tumors in TCGA database.
