# Supplementary material for: Demethylase ALKBH5 suppresses invasion of gastric cancer via PKMYT1 m6A modification
Source: Mol Cancer. 2022 Feb 3;21:34. doi: 10.1186/s12943-022-01522-y (PMC8812266; doi:10.1186/s12943-022-01522-y)
Supplement: Supplementary file 6 — Additional file 6: Table S1. RT-PCR Primer Sequences. [file 12943_2022_1522_MOESM6_ESM.docx]

**Tables with legends.**

Table S1. RT-PCR Primer Sequences

|  | F | R |
| --- | --- | --- |
| HECBPA | CCAAGAAGTCGGTGGACAAGAACA | GTCATTGTCACTGGTCAGCTCCAG |
| PKMYT1 | GTAAAACGACGGCCAGTCAATAAAGGGACAGGAAGGGC | ACACCCAGGTAAGCAGGTCC |
| GAPDH | CAGGAGGCATTGCTGATGAT | GAAGGCTGGGGCTCATTT |
| ALKBH5 | GACAAGGAAGAGAACCGGCG | GCATCTTCACCTTTCGGGCA |
| FTO | CTCTTCCAGCTGTCGGACCTG | ACAACTCCCAGGATGCACCG |
| MYH9 | ACCGAGAAGATCAATCCATC | AGATACTGGATGACCTTCTTG |
| NT5E | GGCTCCTCTCAATCATGCCG | CCAGAACATTTCATCCGTGTGT |
| PXDN | GTCGTGGCCCACCTGACTG | GTGTCGCTGGGAATGCTG |
| IGF2BP3 | ACTATCCAGCACCTCCCACT | ACTGCACGGGAAACCCATAG |
| primer-1 | CGTCCCACCACTGAGGCT | GCACCTTGAAGACCTCTCCG |
| primer-2 | AAGCGTTCCATGTCACCATTCCG | TGCTGCCCCACCTTCTCGTG |
| primer-3 | CCTGCAGCAACACTGTGAGG | CAGGAAGATGTTGGCAGGCT |
| primer-4 | TGTCAAGCCTGCCAACATCTTCC | TACCCAGCTCCACCAGCAGTC |
| primer-5 | GACAGCAGCCTCTCCAGCAAC | TGTCACTTAGGTCCAGGGCATCC |
