# Supplementary material for: Demethylase ALKBH5 suppresses invasion of gastric cancer via PKMYT1 m6A modification
Source: Mol Cancer. 2022 Feb 3;21:34. doi: 10.1186/s12943-022-01522-y (PMC8812266; doi:10.1186/s12943-022-01522-y)
Supplement: Supplementary file 7 — Additional file 7. The specific sequence of CDS and mut plasmids in PKMYT1. [file 12943_2022_1522_MOESM7_ESM.docx]

CDS区

Atgctagaacgctg gcaccagctg cagccccggc gggtgtcatt ccggggcgag gcctcagagactctgcagag ccctgggtat gacccaagcc ggccagagtc cttcttccag cagagcttccagaggctcag ccgcctgggc catggctcct acggagaggt cttcaaggtg cgctccaaggaggacggccg gctctatgcg gtaaagcgtt ccatgtcacc attccggggc cccaaggaccgggcccgcaa gttggccgag gtgggcagcc acgagaaggt ggggcagcac ccatgctgcgtgcggctgga gcaggcctgg gaggagggcg gcatcctgta cctgcagacg gagctgtgcgggcccagcct gcagcaacac tgtgaggcct ggggtgccag cctgcctgag gcccaggtctggggctacct gcgggacacg ctgcttgccc tggcccatct gcacagccag ggcctggtgcaccttgatgt caagcctgcc aacatcttcc tggggccccg gggccgctgc aagctgggtgacttcggact gctggtggag ctgggtacag caggagctgg tgaggtccag gagggagacccccgctacat ggcccccgag ctgctgcagg gctcctatgg gacagcagcg gatgtgttcagtctgggcct caccatcctg gaagtggcat gcaacatgga gctgccccac ggtggggagggctggcagca gctgcgccag ggctacctgc cccctgagtt cactgccggt ctgtcttccgagctgcgttc tgtccttgtc atgatgctgg agccagaccc caagctgcgg gccacggccgaggccctgct ggcactgcct gtgttgaggc agccgcgggc ctggggtgtg ctgtggtgcatggcagcgga ggccctgagc cgagggtggg ccctgtggca ggccctgctt gccctgctctgctggctctg gcatgggctg gctcaccctg ccagctggct acagcccctg ggcccgccagccaccccgcc tggctcacca ccctgcagtt tgctcctgga cagcagcctc tccagcaactgggatgacga cagcctaggg ccttcactct cccctgaggc tgtcctggcc cggactgtggggagcacctc caccccccgg agcaggtgca cacccaggga tgccctggac ctaagtgacatcaactcaga gcctcctcgg ggctccttcc cctcctttga gcctcggaac ctcctcagcctgtttgagga caccctagac ccaacctga

Mut-1

ATGCTAGAACGCTGGCACCAGCTGCAGCCCCGGCGGGTGTCATTCCGGGGCGAGGCCTCAGAGGCTCTGCAGAGCCCTGGGTATGACCCAAGCCGGCCAGAGTCCTTCTTCCAGCAGAGCTTCCAGAGGCTCAGCCGCCTGGGCCATGGCTCCTACGGAGAGGTCTTCAAGGTGCGCTCCAAGGAGGACGGCCGGCTCTATGCGGTAAAGCGTTCCATGTCACCATTCCGGGGCCCCAAGGACCGGGCCCGCAAGTTGGCCGAGGTGGGCAGCCACGAGAAGGTGGGGCAGCACCCATGCTGCGTGCGGCTGGAGCAGGCCTGGGAGGAGGGCGGCATCCTGTACCTGCAGACGGAGCTGTGCGGGCCCAGCCTGCAGCAACACTGTGAGGCCTGGGGTGCCAGCCTGCCTGAGGCCCAGGTCTGGGGCTACCTGCGGGACACGCTGCTTGCCCTGGCCCATCTGCACAGCCAGGGCCTGGTGCACCTTGATGTCAAGCCTGCCAACATCTTCCTGGGGCCCCGGGGCCGCTGCAAGCTGGGTGACTTCGGACTGCTGGTGGAGCTGGGTACAGCAGGAGCTGGTGAGGTCCAGGAGGGAGACCCCCGCTACATGGCCCCCGAGCTGCTGCAGGGCTCCTATGGGACAGCAGCGGATGTGTTCAGTCTGGGCCTCACCATCCTGGAAGTGGCATGCAACATGGAGCTGCCCCACGGTGGGGAGGGCTGGCAGCAGCTGCGCCAGGGCTACCTGCCCCCTGAGTTCACTGCCGGTCTGTCTTCCGAGCTGCGTTCTGTCCTTGTCATGATGCTGGAGCCAGACCCCAAGCTGCGGGCCACGGCCGAGGCCCTGCTGGCACTGCCTGTGTTGAGGCAGCCGCGGGCCTGGGGTGTGCTGTGGTGCATGGCAGCGGAGGCCCTGAGCCGAGGGTGGGCCCTGTGGCAGGCCCTGCTTGCCCTGCTCTGCTGGCTCTGGCATGGGCTGGCTCACCCTGCCAGCTGGCTACAGCCCCTGGGCCCGCCAGCCACCCCGCCTGGCTCACCACCCTGCAGTTTGCTCCTGGACAGCAGCCTCTCCAGCAACTGGGATGACGACAGCCTAGGGCCTTCACTCTCCCCTGAGGCTGTCCTGGCCCGGACTGTGGGGAGCACCTCCACCCCCCGGAGCAGGTGCACACCCAGGGATGCCCTGGACCTAAGTGACATCAACTCAGAGCCTCCTCGGGGCTCCTTCCCCTCCTTTGAGCCTCGGAACCTCCTCAGCCTGTTTGAGGACACCCTAGACCCAACCTGA

Mut-2

ATGCTAGAACGCTGGCACCAGCTGCAGCCCCGGCGGGTGTCATTCCGGGGCGAGGCCTCAGAGACTCTGCAGAGCCCTGGGTATGACCCAAGCCGGCCAGAGTCCTTCTTCCAGCAGAGCTTCCAGAGGCTCAGCCGCCTGGGCCATGGCTCCTACGGAGAGGTCTTCAAGGTGCGCTCCAAGGAGGACGGCCGGCTCTATGCGGTAAAGCGTTCCATGTCACCATTCCGGGGCCCCAAGGGCCGGGCCCGCAAGTTGGCCGAGGTGGGCAGCCACGAGAAGGTGGGGCAGCACCCATGCTGCGTGCGGCTGGAGCAGGCCTGGGAGGAGGGCGGCATCCTGTACCTGCAGACGGAGCTGTGCGGGCCCAGCCTGCAGCAACACTGTGAGGCCTGGGGTGCCAGCCTGCCTGAGGCCCAGGTCTGGGGCTACCTGCGGGACACGCTGCTTGCCCTGGCCCATCTGCACAGCCAGGGCCTGGTGCACCTTGATGTCAAGCCTGCCAACATCTTCCTGGGGCCCCGGGGCCGCTGCAAGCTGGGTGACTTCGGACTGCTGGTGGAGCTGGGTACAGCAGGAGCTGGTGAGGTCCAGGAGGGAGACCCCCGCTACATGGCCCCCGAGCTGCTGCAGGGCTCCTATGGGACAGCAGCGGATGTGTTCAGTCTGGGCCTCACCATCCTGGAAGTGGCATGCAACATGGAGCTGCCCCACGGTGGGGAGGGCTGGCAGCAGCTGCGCCAGGGCTACCTGCCCCCTGAGTTCACTGCCGGTCTGTCTTCCGAGCTGCGTTCTGTCCTTGTCATGATGCTGGAGCCAGACCCCAAGCTGCGGGCCACGGCCGAGGCCCTGCTGGCACTGCCTGTGTTGAGGCAGCCGCGGGCCTGGGGTGTGCTGTGGTGCATGGCAGCGGAGGCCCTGAGCCGAGGGTGGGCCCTGTGGCAGGCCCTGCTTGCCCTGCTCTGCTGGCTCTGGCATGGGCTGGCTCACCCTGCCAGCTGGCTACAGCCCCTGGGCCCGCCAGCCACCCCGCCTGGCTCACCACCCTGCAGTTTGCTCCTGGACAGCAGCCTCTCCAGCAACTGGGATGACGACAGCCTAGGGCCTTCACTCTCCCCTGAGGCTGTCCTGGCCCGGACTGTGGGGAGCACCTCCACCCCCCGGAGCAGGTGCACACCCAGGGATGCCCTGGACCTAAGTGACATCAACTCAGAGCCTCCTCGGGGCTCCTTCCCCTCCTTTGAGCCTCGGAACCTCCTCAGCCTGTTTGAGGACACCCTAGACCCAACCTGA
