# Supplementary material for: Demethylase ALKBH5 suppresses invasion of gastric cancer via PKMYT1 m6A modification
Source: Mol Cancer. 2022 Feb 3;21:34. doi: 10.1186/s12943-022-01522-y (PMC8812266; doi:10.1186/s12943-022-01522-y)
Supplement: Supplementary file 8 — Additional file 8. PKMYT1 mRNA-SRAMP prediction Results. [file 12943_2022_1522_MOESM8_ESM.pdf]

Your job 'Zvahd9wT3V' is finished

- Submission time: **April 14, 2021, 4:28 a.m.**
- Prediction mode: **full, Generic**
- Analyzing RNA secondary structure: **NO**

Your FASTA sequence:

>NM_001258450.2 Homo sapiens protein kinase, membrane associated tyrosine/threonine 1 (PKMYT1), transcript variant 3, mRNA
GCGCGUUCACGGGCCGUUCCCCCUCACGGGAGUCCUCCGCCCGGGCGUCCGGAACAGUCGACGGCAGACUCCGGCCCGCUGAGCCACCCGAGGG
UCCGCGGACCCGGAAUCUGGGCCCUCGCGGACCCGCGCCCCGCCCAGUCGCCCCAGGGCUUCCCCACACCCACGGAGUGAAGUCAGCCGCGGCCC
AACUUACCGUCUACCGGGAAAGGUGGCCAGCAGAUGUGUCGGGCCUGGUGAGAGGGUGAGGCGAGACGGCCCGAUCGCCCAGGGCCCCGGAAG
ACCCCCGCCUGGCCUUAGCUCAGGGACACCCUGGAUUCACGUGGGAGCCCCUGCUCCUGCCUCCCCCGUCCCACCACUGAGGCUGUUGGGCCAG
UAGAACGCUGGCACCAGCUGCAGCCCCGGCGGGUGUCAUUCCGGGGCGAGGCCUCAGAGACUCUGCAGAGCCCUGGGUAUGACCCAAGCCGGCC
UCCAGCAGAGCUUCCAGAGGCUCAGCCGCCUGGGCCAUGGCUCCUACGGAGAGGUCUUCAAGGUGCGCUCCAAGGAGGACGGCCGGCUCUAUG
UCCAUGUCACCAUUCCGGGGCCCCAAGGACCGGGCCCGCAAGUUGGCCGAGGUGGGCAGCCACGAGAAGGUGGGGCAGCACCCAUGCUGCGUG
GCCUGGGAGGAGGGCGGCAUCCUGUACCUGCAGACGGAGCUGUGCGGGCCCAGCCUGCAGCAACACUGUGAGGCCUGGGGUGCCAGCCUGCCU
CUGGGGCUACCUGCGGGACACGCUGCUUGCCCUGGCCCAUCUGCACAGCCAGGGCCUGGUGCACCUUGAUGUCAAGCCUGCCAACAUCUUCCUG
CCGCUGCAAGCUGGGUGACUUCGGACUGCUGGUGGAGCUGGGUACAGCAGGAGCUGGUGAGGUCCAGGAGGGAGACCCCCGCUACAUGGCCCC
AGGGCUCCUAUGGGACAGCAGCGGAUGUGUUCAGUCUGGGCCUCACCAUCCUGGAAGUGGCAUGCAACAUGGAGCUGCCCCACGGUGGGGAGG
CUGCGCCAGGGCUACCUGCCCCCUGAGUUCACUGCCGGUCUGUCUUCCGAGCUGCGUUCUGUCCUUGUCAUGAUGCUGGAGCCAGACCCCAAG
GCCGAGGCCCUGCUGGCACUGCCUGUGUUGAGGCAGCCGCGGGCCUGGGGUGUGCUGUGGUGCAUGGCAGCGGAGGCCCUGAGCCGAGGGUG
GGCCCUGCUUGCCCUGCUCUGCUGGCUCUGGCAUGGGCUGGCUCACCCUGCCAGCUGGCUACAGCCCCUGGGCCCGCCAGCCACCCCGCCUGGC
AGUUUGCUCCUGGACAGCAGCCUCUCCAGCAACUGGGAUGACGACAGCCUAGGGCCUUCACUCUCCCCUGAGGCUGUCCUGGCCCGGACUGUG
ACCCCCCGGAGCAGGUGCACACCCAGGGAUGCCCUGGACCUAAGUGACAUCAACUCAGAGCCUCCUCGGGGCUCCUUCCCCUCCUUUGAGCCUC
GCCUGUUUGAGGACACCCUAGACCCAACCUGAGCCCCAGACUCUGCCUCUGCACUUUUAACCUUUUAUCCUGUGUCUCUCCCGUCGCCCUUGAA
CUCGGGAACUCCCAUGGUCUUCUCUGCCUGGCCGUGUCUAAUAAAAAGUAUUUGAACCUUGGGAGCACCCAA

Please find your results below. You may also download the results in plain text from here. **Note that the results will be automatically deleted after 72 hours!**

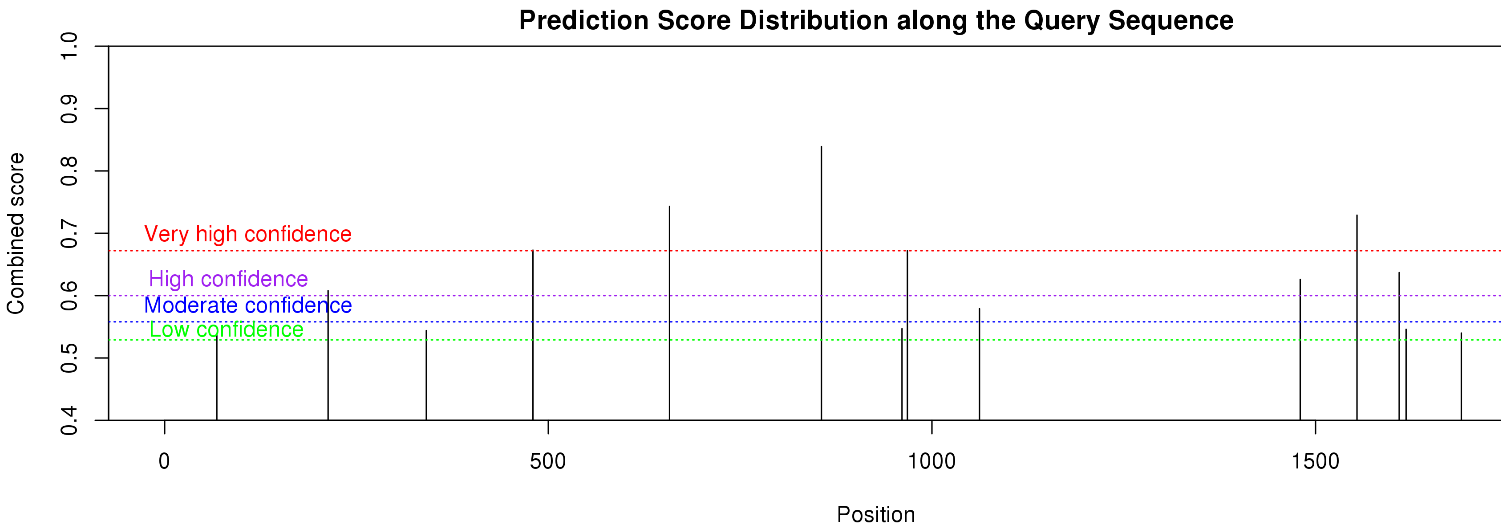

#	Position	Sequence context	Structural context	Local structure visualization	Score(binary)	Score(knn)	Score(spectrum)	Score(combined)	Decision
1	68	CGUCC GGAAC AGUCG ACGGC AGACU CCGGC CCGCU GAGCC ACCCG	N/A	N/A	0.554	0.697	0.492	0.536	m6A site (**Low** confidence)
2	213	GCCGC GGCCC UGCCU GGGAG GAACU UACCG UCUAC CGGGA AAGGU	N/A	N/A	0.675	0.808	0.490	0.608	m6A site (**High** confidence)
3	341	CCCGC CUGGC CUUAG CUCAG GGACA CCCUG GAUUC ACGUG GGAGC	N/A	N/A	0.599	0.757	0.443	0.544	m6A site (**Low** confidence)
4	480	AUUCC GGGGC	N/A	N/A	0.731	0.754	0.583	0.673	m6A site

		GAGGC CUCAG AGACU CUGCA GAGCC CUGGG UAUGA							(Very high confidence)
5	658	UCACC AUUCC GGGGC CCCAA GGACC GGGCC CGCAA GUUGG CCGAG	N/A	N/A	0.842	0.787	0.601	0.743	m6A site (Very high confidence)
6	856	CAGGU CUGGG GCUAC CUGCG GGACA CGCUG CUUGC CCUGG CCCAU	N/A	N/A	0.843	0.711	0.851	0.839	m6A site (Very high confidence)
7	961	CGGGG CCGCU GCAAG CUGGG UGACU UCGGA CUGCU GGUGG AGCUG	N/A	N/A	0.505	0.692	0.586	0.547	m6A site (Low confidence)
8	968	GCUGC AAGCU GGGUG ACUUC GGACU GCUGG UGGAG CUGGG UACAG	N/A	N/A	0.702	0.713	0.626	0.672	m6A site (Very high confidence)
9	1062	GCUGC UGCAG GGCUC CUAUG GGACA GCAGC GGAUG UGUUC AGUCU	N/A	N/A	0.591	0.618	0.558	0.579	m6A site (Moderate confidence)
10	1480	CCACC CUGCA GUUUG CUCCU GGACA GCAGC CUCUC CAGCA ACUGG	N/A	N/A	0.645	0.843	0.572	0.626	m6A site (High confidence)
11	1554	CCCUG AGGCU GUCCU GGCCC GGACU GUGGG GAGCA CCUCC ACCCC	N/A	N/A	0.761	0.840	0.670	0.729	m6A site (Very high confidence)
12	1609	UGCAC ACCCA GGGAU GCCCU GGACC UAAGU GACAU CAACU CAGAG	N/A	N/A	0.625	0.766	0.639	0.637	m6A site (High confidence)
13	1618	AGGGA UGCCC UGGAC CUAAG UGACA UCAAC UCAGA GCCUC CUCGG	N/A	N/A	0.476	0.619	0.634	0.546	m6A site (Low confidence)
14	1690	AACCU CCUCA GCCUG UUUGA GGACA CCCUA GACCC AACCU GAGCC	N/A	N/A	0.592	0.465	0.478	0.540	m6A site (Low confidence)
